# An iterative process produces oxamniquine derivatives that kill the major species of schistosomes infecting humans

**DOI:** 10.1371/journal.pntd.0008517

**Published:** 2020-08-18

**Authors:** Meghan A. Guzman, Anastasia R. Rugel, Reid S. Tarpley, Sevan N. Alwan, Frédéric D. Chevalier, Dmytro P. Kovalskyy, Xiaohang Cao, Stephen P. Holloway, Timothy J. C. Anderson, Alexander B. Taylor, Stanton F. McHardy, Philip T. LoVerde

**Affiliations:** 1 Departments of Biochemistry and Structural Biology, the University of Texas Health Science Center, San Antonio, Texas, United States of America; 2 Pathology and Laboratory Medicine, the University of Texas Health Science Center, San Antonio, Texas, United States of America; 3 Center for Innovative Drug Discovery, Department of Chemistry, University of Texas at San Antonio, San Antonio, Texas, United States of America; 4 Program in Host-Pathogen Interactions, Texas Biomedical Research Institute, San Antonio, Texas, United States of America; 5 Program in Disease Intervention and Prevention, Texas Biomedical Research Institute, San Antonio, Texas, United States of America; 6 X-ray Crystallography Core Laboratory, Institutional Research Cores, University of Texas Health Science Center, San Antonio, Texas, United States of America; University of Pennsylvania, UNITED STATES

## Abstract

Currently there is only one method of treatment for human schistosomiasis, the drug praziquantel. Strong selective pressure has caused a serious concern for a rise in resistance to praziquantel leading to the necessity for additional pharmaceuticals, with a distinctly different mechanism of action, to be used in combination therapy with praziquantel. Previous treatment of *Schistosoma mansoni* included the use of oxamniquine (OXA), a prodrug that is enzymatically activated in *S*. *mansoni* but is ineffective against *S*. *haematobium* and *S*. *japonicum*. The oxamniquine activating enzyme was identified as a *S*. *mansoni* sulfotransferase (*Sm*SULT-OR). Structural data have allowed for directed drug development in reengineering oxamniquine to be effective against *S*. *haematobium* and *S*. *japonicum*. Guided by data from X-ray crystallographic studies and *Schistosoma* worm killing assays on oxamniquine, our structure-based drug design approach produced a robust SAR program that tested over 300 derivatives and identified several new lead compounds with effective worm killing *in vitro*. Previous studies resulted in the discovery of compound CIDD-0066790, which demonstrated broad-species activity in killing of schistosome species. As these compounds are racemic mixtures, we tested and demonstrate that the R enantiomer CIDD-007229 kills *S*. *mansoni*, *S*. *haematobium* and *S*. *japonicum* better than the parent drug (CIDD-0066790). The search for derivatives that kill better than CIDD-0066790 has resulted in a derivative (CIDD- 149830) that kills 100% of *S*. *mansoni*, *S*. *haematobium* and *S*. *japonicum* adult worms within 7 days. We hypothesize that the difference in activation and thus killing by the derivatives is due to the ability of the derivative to fit in the binding pocket of each sulfotransferase (*Sm*SULT-OR, *Sh*SULT-OR, *Sj*SULT-OR) and to be efficiently sulfated. The purpose of this research is to develop a second drug to be used in conjunction with praziquantel to treat the major human species of *Schistosoma*. Collectively, our findings show that CIDD-00149830 and CIDD-0072229 are promising novel drugs for the treatment of human schistosomiasis and strongly support further development and *in vivo* testing.

## Author summary

Schistosomiasis affects more than 229 million people in 78 countries of the world. The main treatment is Mass Drug Administration with praziquantel. With donations to the World Health Organization, approximately 250 million tablets of praziquantel are being administered in sub-Saharan Africa where about 90% of the cases of schistosomiasis occur. The concern with a monotherapy is the development of drug resistance. The need for new drugs with a different mode of action to be used in combination with praziquantel is great. In this regard, we have taken oxamniquine, a drug that was previously used to treat *Schistosoma mansoni* but was ineffective against *S*. *haematobium* and *S*. *japonicum*, and determined the enzyme responsible for activation of oxamniquine as a sulfotransferase. With this knowledge and the sulfotransferase crystal structure, we were able to determine the mode of action of the drug and develop an iterative approach of soaking oxamniquine derivatives into sulfotransferase crystals, determining structure function relationships, synthesizing new derivatives and testing them in an *in vitro* killing assay. The most effective derivatives are soaked into new crystals and the process repeated. We have identified two derivatives, CIDD-0072229 and CIDD-149830 that will kill *S*. *haematobium* and *S*. *japonicum* in addition to *S*. *mansoni*. CIDD-149830 will kill 100% of the worms of all three species within 7 days.

## Introduction

Human schistosomiasis is caused by three major species: *S*. *mansoni*, *S*. *haematobium*, and *S*. *japonicum*. Current estimates indicate that globally schistosomiasis affects over 229 million people in 78 countries [1–3]. Of those infected, over 100 million are estimated to be symptomatic, 20 million experience long term complications due to infection, and anywhere from 20,000–200,000 people are estimated to die from the disease annually [4–6]. Furthermore, these three major species account for the vast majority of global burden [7–10]. Currently, there is no effective vaccine against human schistosomiasis; however, there is a drug that is effective against all three major human schistosome species, praziquantel (PZQ). The mainstay of schistosome control programs have used repeated mass chemotherapy with PZQ–currently 250 million doses per annum–of the at-risk and infected populations of human hosts [5, 11–13].

Previous treatments for *S*. *mansoni* include oxamniquine (OXA) and hycanthone (HYC). OXA was used extensively in Brazil [14, 15] until PZQ usage became more prevalent [12, 16]. OXA is only effective against the adult worm stage of *S*. *mansoni*, HYC is effective against the adult worm stage of *S*. *mansoni* and *S*. *haematobium* but has been shown to be a carcinogen [17–19] and thus has fallen out of use. Genetic cross studies have indicated that mutations in a single gene are responsible for both HYC and OXA resistance [20] which has developed in the field [21, 22] and has been selected for in the laboratory [23]. The mechanism of OXA activity and the mechanism for OXA resistance were identified by further genetic and crystallographic studies [24, 25]. OXA and HYC are prodrugs that are enzymatically activated in the parasite [24–28]. OXA binds to a specific *S*. *mansoni* sulfotransferase, known as *Sm*SULT-OR, where it is transiently sulfated. The sulfur group on the released sulfate ester undergoes a nucleophilic attack by schistosome macromolecules. In an S_N_2-like reaction, activated OXA forms adducts with DNA and other macromolecules, resulting in killing of the worms [24, 25]. This affects both adult sexes but mainly the males, causing the parasites to detach from hepatoportal circulation and move into the liver where they are eliminated, in part, by the formation of an adduct on DNA thus blocking DNA replication and transcription [25, 27, 29]. If the female worms are not killed, the lack of male worms causes the female worms to revert to an immature state and cease producing eggs [30].

Constant selective pressure through mass chemotherapy has yielded evidence of resistance to PZQ in both the field [31–33] and lab populations [34, 35]. A second drug, to be used in conjunction with PZQ is highly desirable. The ultimate goal is to design an OXA derivative, using directed drug development, which is effective against all major human schistosome species and can be used in combination with PZQ to combat emerging resistance and improve overall treatment efficacy. Herein, we report the design, synthesis and *in vitro* evaluation of novel analogs of OXA, lead compounds which are shown to be efficacious against all three human species of *Schistosoma*.

## Materials and methods

### Ethics

This study was performed in strict accordance with the University of Texas Health Science Center San Antonio Office of the Institutional Animal Care and Use Committee (UTHSCSA IACUC). Research involving the use of animals conducted at UTHSCSA are in accordance with Federal, State and local rules and regulations. Animals were euthanized in accordance with IACUC protocol (University of Texas Health IACUC Protocol #08039) by intraperitoneal injection using Fatal-Plus (Butler Animal Health, Ohio), a sodium pentobarbital solution, with 10% heparin added.

### Parasite maintenance

Strains of schistosome parasites: *S*. *mansoni* LE, *S*. *haematobium* Egyptian and *S*. *japonicum* Chinese were maintained by passage through species-specific snail intermediate hosts (*Biomphalaria glabrata*, *Bulinus truncatus*, and *Oncomelania hupensis*, respectively) and Golden Syrian Hamsters as a definitive host. Definitive hosts were infected with 250–1000 cercariae, for parasite maintenance. *S*. *japonicum* exposed animals received 250 cercariae, *S*. *haematobium* 500 cercariae and *S*. *mansoni* up to 1000 cercariae. Infected *Oncomelania* snails were provided by BRI via the NIAID schistosomiasis resource center under NIH-NIAID Contract No. HHSN272201000005I.

### Adult parasite recovery

Definitive host animals were sacrificed 45-days post-infection for *S*. *mansoni* and *S*. *japonicum*. For *S*. *haematobium*, hamsters were sacrificed at day 90 post infection. The adult parasites were flushed by perfusion as previously described [36] using 0.9% saline containing EDTA. *S*. *haematobium* adult worms required additional manual dissection from the mesenteries and fat deposits along the large intestine.

### Adult worm *in vitro* culture

Adult worms were cultured in 1ml 1X Dulbecco's Modified Eagle Medium (DMEM, Gibco) with 10% Heat Inactivated Fetal Bovine Serum (FBS, Atlantic Biologicals) and 1X antibiotic/antimycotic (Ab/Am, GIBCO). Worms were manually sorted by sex under a dissecting stereomicroscope and aliquoted to 10 worms per well in a 24-well plate. Worm pairs were placed 10 couples per well as above. Worms were kept in an incubator at 37°C and 5% CO_2_. Media was changed every 2–3 days.

### Compound design *and* OXA derivatives

Oxamniquine derivatives were designed and synthesized by the Center for Innovative Drug Discovery (CIDD) utilizing a structure-based drug design approach using X-ray crystallographic structural data and structure-activity relationship data based on the schistosomicidal efficacy of the derivatives *in vitro*. The synthesis of the CIDD-0072229 chemical series was previously published [37]. The synthesis used to access CIDD-0149830, along with all supporting analytical data is provided in the supporting information (S1 Table). An iterative process was used to develop new drugs. The derivatives that were more effective than OXA were soaked into *Sm*SULT-OR crystals, the CIDD used that information to synthesize new derivatives that were tested for schistosomicidal activity in an *in vitro* killing assay. The OXA derivatives that showed the best killing were soaked into new crystals and the process repeated.

### OXA derivative *in vitro* screen

Derivatives were solubilized in 100% Dimethyl sulfoxide (DMSO) and diluted to working concentration of 50 mM and added directly to each well within 2–24 hours after harvesting adult schistosomes from the hamsters at a final concentration of 143 μM. As each derivative has a different molecular mass, this allowed a direct comparison between derivatives. Each derivative was tested in triplicate allowing for up to eight variables per plate. We performed a dose-dependent killing response using 143 μM, 71.5 μM and 35.75 μM. As the derivatives were all racemic mixtures, we tested the enantiomers of the best derivative at that time, CIDD-0066790 (compound 12a in [37]), for schistosomicidal activity. DMSO, OXA or HYC were used at 143 μM as controls as needed. Drugs were incubated with adult male, female worms or worm pairs at 37°C, 5% CO_2_ for 45 minutes, mimicking physiological conditions [25]. The worms were washed with plain media 3 times to remove any residual derivatives. Worms were then incubated in culture media as previously described for a period of up to 14 days. Worm motility, opaque color, shedding and tegument blebbing were used as an assessment of survival and death/morbidity. OXA was the positive control for *S*. *mansoni* and HYC for *S*. *haematobium*. These are the standards against which we measured the efficacy of the modified OXA. *S*. *japonicum* does not have a positive control. Worm killing was assessed by observing plates daily for up to 14 days under a stereomicroscope and then counting the number of dead/morbid worms. Sensitive parasites typically start dying by day 7 and are all dead by day 14.

### Quantitative real time PCR

Whole RNA was obtained from frozen samples of adult *S*. *mansoni* worms. RNA was extracted and purified according to manufacturer instructions for total RNA isolation (Molecular Research Center Inc.). cDNA was synthesized using qScript cDNA Supermix (Quanta Biosciences) according to manufacturer instructions from 1 μg whole RNA for a final concentration of 50 μg/μl cDNA in nuclease free water.

Quantitative Real Time PCR (qRT-PCR) was used to determine the relative quantities of sulfotransferase transcribed between adult male and female schistosomes. 150 μg of cDNA was used for relative quantification with gene specific primers and iTaq Universal SYBR Green Supermix (BioRad) containing hot-start iTaq DNA polymerase, dNTPs, MgCl2, SYBR Green I dye, and ROX reference dye. Primers were designed using PerlPrimer v1.21 (Smp_089320_qF1 ATTGGATGGTTACATAGCAACTAC, qR1 CCATGGATCATTTGATTTGGGT) and assayed for efficiency at 1:0, 1:5, 1:25, and 1:100 cDNA concentrations. GAPDH was used as an endogenous control. The primers for SmGAPDH are qF1 GTGAAAGAGATCCAGCAAACAT and qR1 ATATGAGCCTGAGCTTTATCAATG. The qRT-PCR reaction was performed in 10 μL reaction and contained 5μl iTaq Universal SYBR Green Supermix (BioRad), 3 μl cDNA (50μg/μl), 1 μl each of forward and reverse primers 100 μM. The qRT-PCR profile was 50ºC for 2 min, 95ºC for 10 min, 40 cycles of 95ºC for 15 s, 60ºC for 1 min, and a final step of 60ºC for 5 min (Applied Biosystems 7500 FastReal-Time PCR System). Each experiment was performed in triplicate.

### Computational modeling studies

All modeling studies were performed with the Schrӧdinger suite (version 2015–4) [38] except molecular dynamics simulations that were performed using GROMACS 5.1.2 [39]. Molecular visualization was performed with Chimera, Maestro and VMD [38, 40, 41]. The crystal structure of *Sj*SULT-OR (PDB entry 5TIZ) contains a domain-swapped C-terminal α helix from an adjacent molecule thought to be an artifact [24]; therefore, a homology model was prepared for use in simulations. *Sj*SULT-OR was modeled with the Schrӧdinger Prime module using the crystal structure of *Sm*SULT-OR (PDB entry 4MUA) as a template structure. *Sj*SULT-OR is 52% identical and 69% similar to *Sm*SULT-OR [42].

### Modelling of the CIDD-0072229 interaction with SULT-OR

Compound CIDD-0066790 (compound **12a** in [37] has a stereo center at the C3 carbon of the piperidine ring. We modelled SULT-OR interactions with its R-enantiomer, CIDD-0072229, since it had shown maximal worm killing activity. The plausible complexes were generated using the docking engine Glide [43]. Depending on the conformation of the piperidine ring, CIDD-0072229 can have substantially diverse shapes ranging from linear to compact. We used coordinates of **11g** - *Sm*SULT-OR complex (PDB entry 6MFE). To generate a grid, the crystal structure was first subjected to the Protein Preparation Wizard and a cube for the grid was created with 20 Å distances from the ligand center of mass. The PAP co-substrate was retained during the grid generation and all water molecules were removed but those that make direct hydrogen bonds with the ligand. The extra precision routine was used and up to three states per ligand were allowed to be saved. The hydroxyl group of Tyr154 was allowed to rotate. The top scoring pose was used to model complexes with *Sh*SULT-OR and *Sj*SULT-OR. *Sh*SULT-OR is 70.6% identical and 80.9% similar to *Sm*SULT-OR. To generate *Sh*SULT-OR:CIDD-0072229 and *Sj*SULT-OR:CIDD-0072229 complexes, the crystal structures of *Sh*SULT-OR (PDB entry 5TIV) and the homology model of *Sj*SULT-OR (see above) were superimposed on the complex *Sm*SULT-OR:CIDD-0072229. The newcomplexes were subjected to iterative energy minimization routines to remove steric clashes. As a result, we generated three models M-229, H-229 and J-229 (referring to the complexes of CIDD-0072229 bound to *Sm*SULT-OR, *Sh*SULT-OR and *Sj*SULT-OR, respectively).

To enumerate possible biologically relevant conformations of CIDD-0072229, the ConfGen utility from the Schrӧdinger suite was applied [43]. A library of 63 conformers was then superimposed on the conformation derived from docking and their RMSD values were calculated.

To model CIDD-0149830, an iterative approach was applied. First, we designed a set of CIDD-0066790 derivatives with modification in the central saturated ring while keeping the aromatic moieties intact. Docking to the *Sm*SULT-OR was applied similarly to CIDD-0072229. The best scored ligand was a 3,3’-disubstituted pyrrolidine, which was used to design CIDD-0149830. The compound was then docked into *Sm*SULT-OR using coordinates of CIDD-000206 (compound 9f in [37]) complexed with *Sm*SULT-OR (PDB Entry 6BDR).

### Statistical methods

Survival tests and curves were generated using a custom R script implementing the survival package [44] or Prism (version 8). Differences in the survival function of different treatments were tested using a log-rank test. Multiple pairwise comparisons were corrected using Bonferroni correction.

## Results

### OXA derivative *in vitro* screen

**Fig 1:**
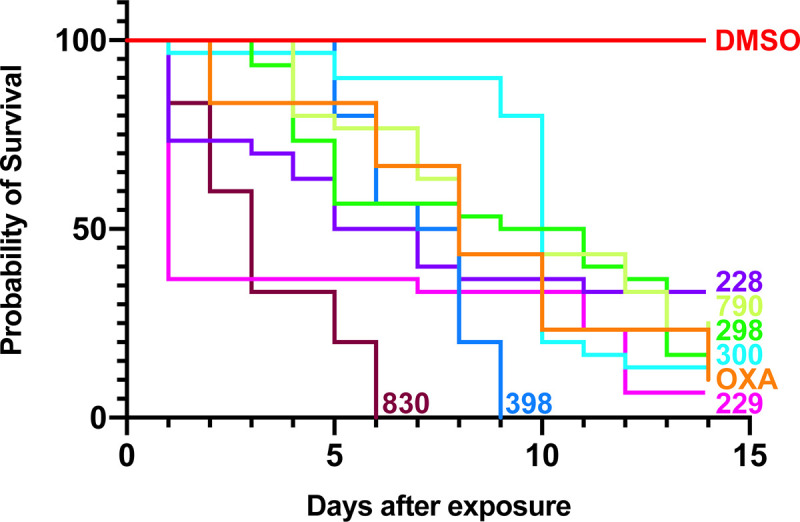
OXA Derivative Screen against *S*. *mansoni in vitro*. OXA derivatives were tested against adult male *S*. *mansoni* worms *in vitro*. All derivatives were solubilized in 100% DMSO and administered at a final concentration of 143 μM per well. All screens were performed in experimental and biological triplicate. Survival was plotted as a percentage over time using the Kaplan-Meier curves. Pair wise comparison was performed using a log-rank test with Bonferroni correction for multiple testing. The p-value threshold for each derivative compared to DMSO was <0.001.

Compounds derived from the structural data of OXA in the *Sm*SULT-OR active site were designed, synthesized and initially tested *in vitro* for worm killing against OXA sensitive *S*. *mansoni* adult male worms [37]. Of >300 OXA derivatives prepared and tested, 16 demonstrated over 70% worm killing against *S*. *mansoni in vitro* ([37], this study). Fig 1 and Table 1 show the *in vitro* killing of *S*. *mansoni*. OXA, the positive control, is denoted by the yellow line and DMSO, the negative control, the red line. All of the derivatives tested showed better killing than OXA. CIDD-007229 (purple line) showed greater than 90% killing and CIDD-72398 (blue line) showed 100% killing by day 9. The most impressive derivative is CIDD-0149830 as it kills 100% of *S*. *mansoni* within 5 days. We employ male worms as they are more sensitive to the drugs. However, CIDD-0149830 will also kill 100% of mature female worms in 4 days but female worms in pairs results in 60% killing in 14 days. Single or paired males die at the same rate (S1 Fig). To address in part the issue of male vs female killing, we performed qRT-PCR to evaluate relative levels of transcripts between male and female adult worms from *S*. *mansoni*. The results indicate that there are relatively higher levels, nearly 5x, of *Sm*SULT-OR transcripts in male adult worms compared to female adult worms (S2 Fig). We tested the ability of lower doses to kill schistosome worms. At 71.5 μM 100% of *S*. *mansoni* worms are killed within 13 days. *S*. *haematobium* and *S*. *japonicum* are killed at 60 and 55% over 14 days (S3 Fig).

**Table 1:** Derivatives of oxamniquine that kill schistosome species*.

OXA Derivative	*S*. *mansoni*	*S*. *haematobium*	*S*. *japonicum*
OXA	60±20	0	0
CIDD-0066790	85 ± 15	40	83
CIDD-0072228	66	57	53
CIDD-0072229	93	95	80 ± 5
CIDD-0072518	70	0	0
CIDD-0072298	80	60	0
CIDD-0072300	85	0	0
CIDD-0072398	100	0	0
CIDD-00149830	100	100	100

* Each of the derivatives were tested in triplicate with 10 male worms per well. Number equals % killing over a 14 day period

As the derivative compounds were racemic compounds, we then tested the enantiomers of one (CIDD-0066790) that showed killing against all 3 schistosome species (Table 1). The CIDD-0066790 enantiomers CIDD-0072228 and CIDD-0072229 demonstrated significant killing of adult male *S*. *mansoni* with the R enantiomer CIDD-0072229 showing >90% killing.

**Fig 2:**
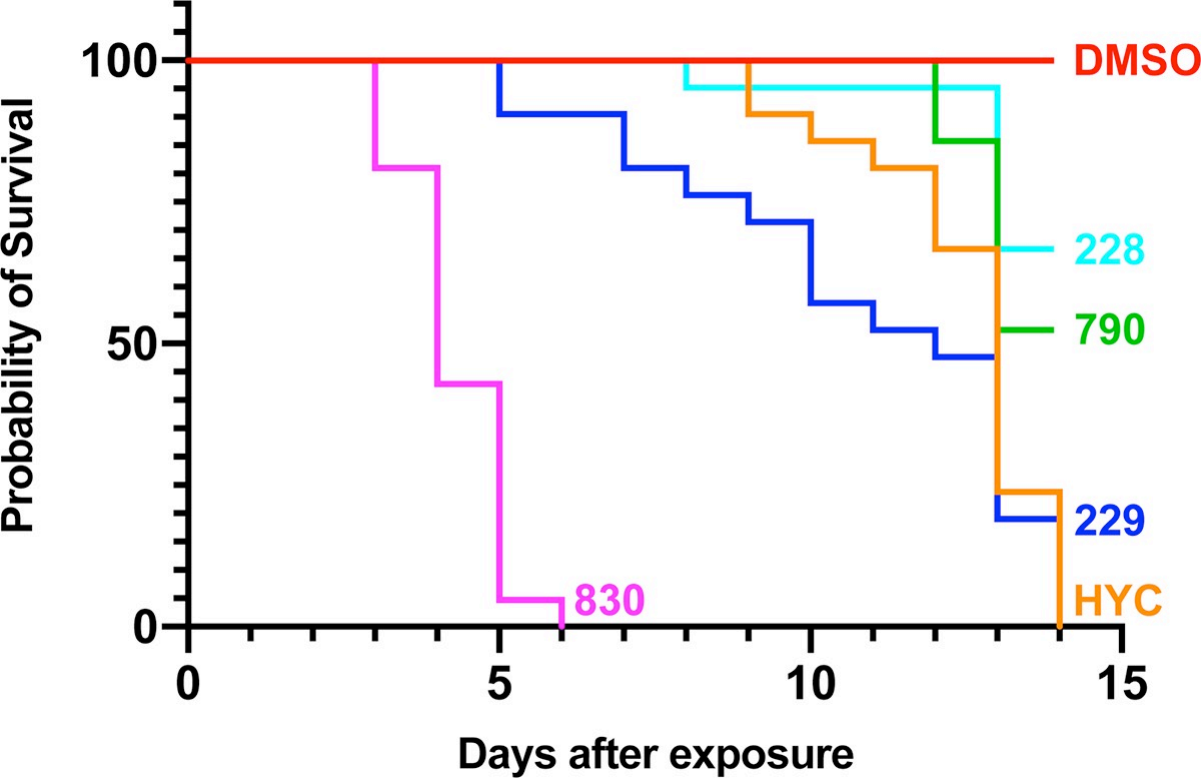
OXA Derivative Screen against *S*. *haematobium in vitro*. OXA derivatives found effective against *S*. *mansoni* were tested against adult male *S*. *haematobium* worms *in vitro*. The derivatives were tested as described in Fig 1 legend. Survival was plotted as a percentage over time using the Kaplan-Meier curves. HYC, hycanthone; CIDD-0066790, a racemic derivative of OXA; CIDD-0072228, *S*-enantiomer of CIDD-0066790; CIDD-0072229, *R*-enantiomer of CIDD-0066790, CIDD-00149830, a racemic derivative of OXA and CIDD-01496830, a racemic derivative of OXA. The p-values for each derivative compared to DMSO was <0.001.

**Fig 3:**
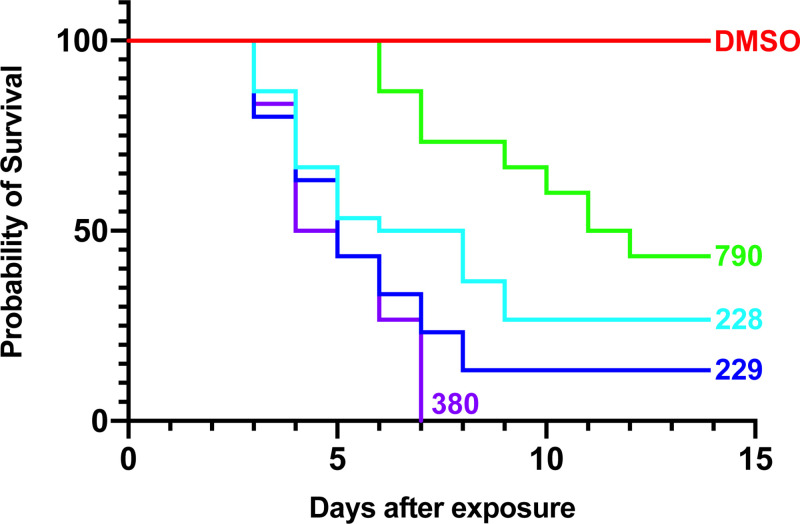
OXA Derivative Screen against *S*. *japonicum in vitro*. OXA derivatives found effective against both *S*. *mansoni* and *S*. *haematobium* were tested against adult male *S*. *japonicum* worms *in vitro*. Figure shows results of CIDD-0066790 and the enantiomers of CIDD-0066790; CIDD-0072228 is the *S*-enantiomer, CIDD-2229 is the *R*-enantiomer and CIDD-01496830. The derivatives were tested as described in Fig 1 legend. The p-value thresholds for each derivative compared to DMSO was <0.001.

The OXA derivatives that demonstrated significant killing against *S*. *mansoni* adult male worms were tested against *S*. *haematobium*. CIDD-0149830 showed 100% killing within 6 days. As above, we tested the enantiomers of CIDD-0066790: CIDD-0072228 and CIDD-0072229 for the ability to kill *S*. *haematobium*. The R*-*enantiomer CIDD-0072229 (blue line) showed >90% killing comparable to hycanthone (orange line), the positive control (Fig 2, Table 1). However, CIDD-0072398 demonstrated no killing of *S*. *haematobium*. CIDD-0149830, CIDD-0066790 and the enantiomers that displayed significant worm killing against both *S*. *mansoni* and *S*. *haematobium in vitro* were tested against *S*. *japonicum* adult male worms *in vitro*. CIDD-0149830 showed 100% killing within 7 days, CIDD-66790 showed over 80% worm killing and the R-enantiomer of CIDD-0066790 showed >90% schistosomicidal activity against *S*. *japonicum in vitro* (Fig 3, Table 1).

**Fig 4:**
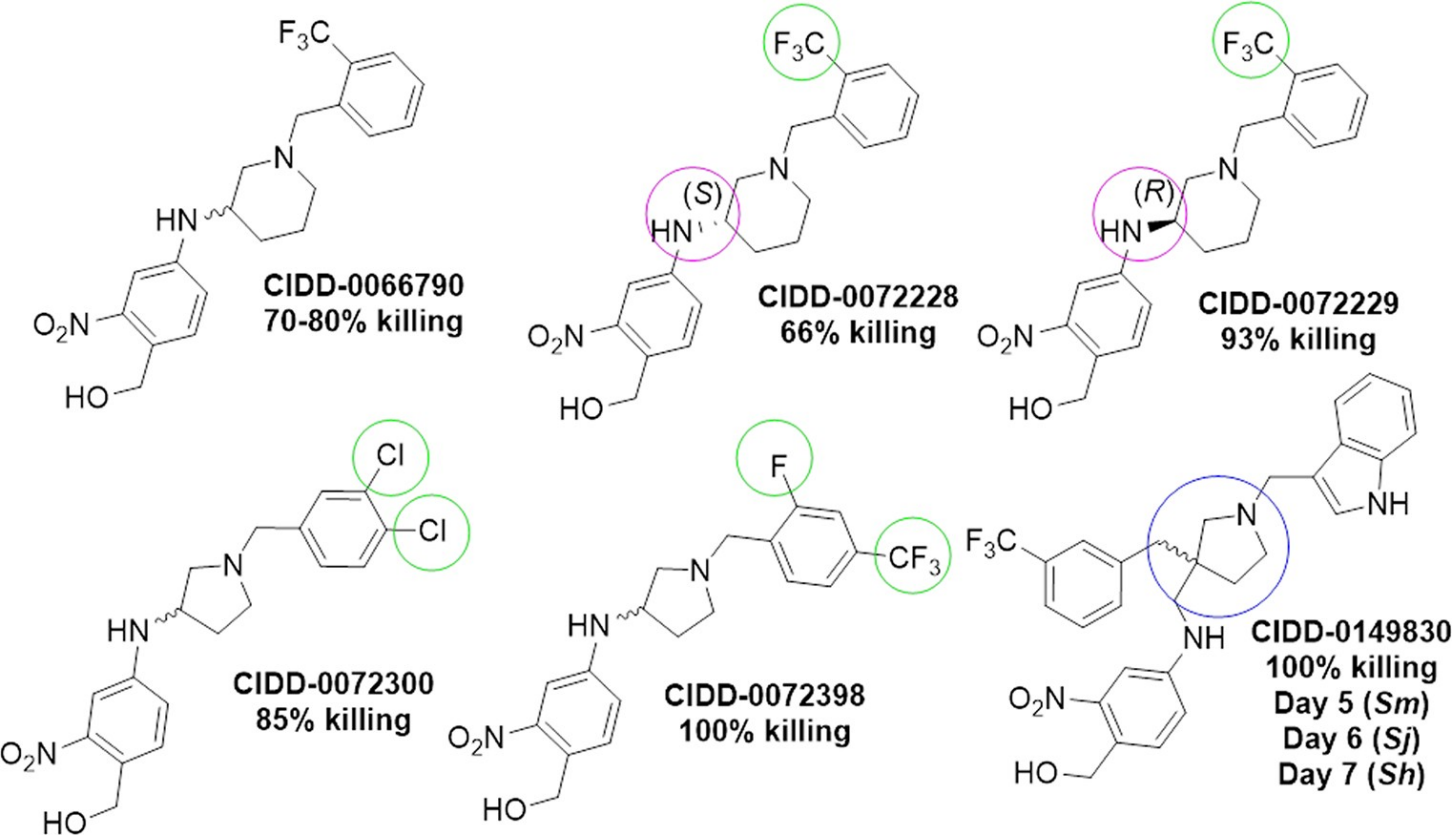
Chemical structure of schistosomicidal OXA derivatives. Key structural modifications highlighted as follows; pink-single enantiomers, green-substitution on benzyl side chain and blue-3,3’-disubstituted pyrrolidine core. % killing refers to the ability of the derivative to kill *S*. *mansoni* male worms. Under CIDD-0149830 the number of days it took the derivative to kill 100% of *S*. *mansoni* (*Sm*), *S*. *haematobium* (*Sh*) and *S*. *Japonicum* (*Sj*) is listed.

Fig 4 shows a comparison of CIDD-0066790, CIDD-0072228 (S*-*enantiomer of CIDD-0066790), CIDD-0072229 (R*-*enantiomer of CIDD-0066790), CIDD-0072300, CIDD-0072398 and CIDD-0149830. The difference in R side groups is boxed. The first five compounds CIDD-0066790, CIDD-0072228, CIDD-0072229, CIDD-0072300 and CIDD-0072398 are from the 3-aminopiperidine or 3-aminopyrrolidine series. CIDD-0066790 and its enantiomers possess the 2-trifluoromethyl substituent on the benzyl sidechain, and only differ by the absolute stereochemical configuration at piperidine C-3 position. CIDD-0072300 and CIDD-0072398 are from the corresponding 3-aminopyrrolidine series and only differ by the nature of the electron-withdrawing substituent on the benzyl ring (i.e. 3, 4-dichloro vs. 2-fluoro-4-trifluoromethyl). Our previous SAR studies on these compounds clearly showed that various electron-withdrawing substituents on the benzyl side chain were preferred for antischistosomal activity [37]. CIDD-0149830 is from the 3, 3-disubstitutied pyrrolidine series, wherein the 3-indole methyl substituent resides on the pyrrolidine nitrogen and the 3-CF_3_-benzyl group is bonded to the C-3 carbon of the pyrrolidine.

### Computational modelling of CIDD-0072229 in the binding pockets of the various schistosome sulfotransferases

**Fig 5:**
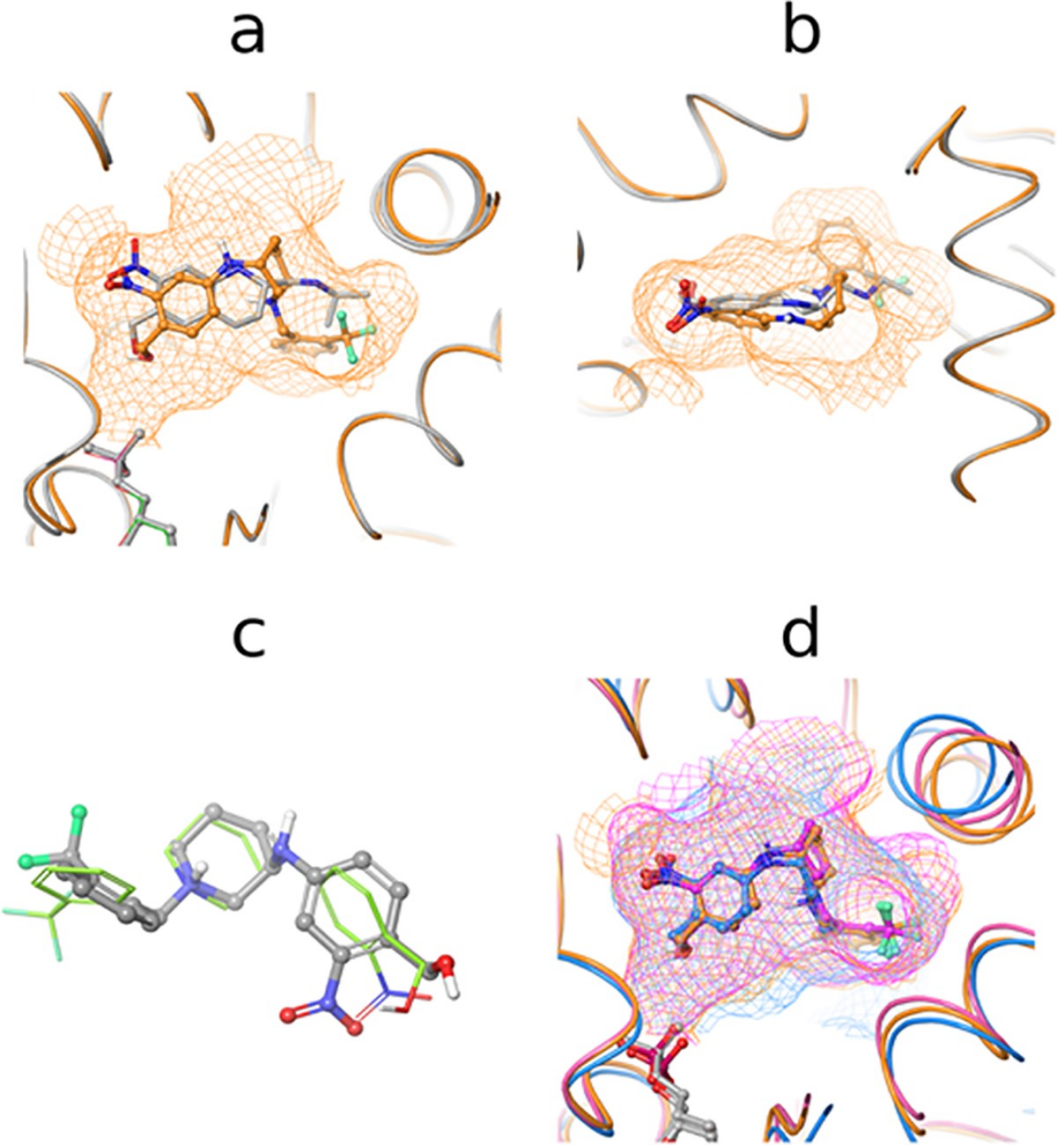
Model of CIDD-0072229 interaction with schistosome SULT-ORs. ***a*** and ***b*.** Model of the complex of *Sm*SULT-OR and CIDD-0072229 superimposed over the crystal structure with the *S*-enantiomer of oxamniquine. Panels ***a*** and ***b*** are rotated 90° about the horizontal plane of the paper with respect to each other. CIDD-0072229 is filling available space forming contacts with residues from α2, α6, α11 and α12 helices. ***c*.** Superposition of the CIDD-0072229 docking pose (in orange ball and sticks) over the low energy conformation generated with the bioactive conformational search method (green sticks). ***d*.** Superposition of models of CIDD-0072229 in complexes with *Sm*SULT-OR (orange), *Sh*SULT-OR (blue) and *Sj*SULT-OR (pink). Shapes of corresponding binding sites are shown as mesh.

Computational modelling was applied to derive a model of the CIDD-0072229 interaction with *Sm*SULT-OR. We used the binding site of *Sm*SULT-OR sourced from the complex with CIDD-000773 [37] (S4 Fig) a structurally similar analog of CIDD-0072229, as a model of the sulfotransferase active site. The top scoring structure generated with docking (docking score -9.985) revealed the extensive interactions between the compound and the active site (S5 Fig). CIDD-0072229 binds within the interior of the active site filling contacts from catalytic residues through α-helices α2, α11 and α12 [24]. Superposition of the complex over the crystal structure with *S*-OXA revealed a nearly complete overlap of CIDD-0072229 over the space occupied by *S*-OXA (Fig 5A and 5B) [45]. In addition, the compound is filling a void between α2 and α12 helices (Fig 5B) forming hydrophobic interactions with Phe39 and Phe43 (S6A Fig).

One of the crucial factors contributing to the potency of the compounds is complementarity of compound conformations in the bound state to their low energy unbound conformation. Such conformations are termed as bioactive. To validate the docking pose of CIDD-0072229, we compared its conformation against a set of conformations generated without a receptor with ConfGen tool [46]. Superposition of the docking conformation with the lowest energy conformation from ConfGen returned a root-mean-square-deviation of 1.2 Å which supports the model (Fig 5C).

Based on the generated model of CIDD-0072229 bound to *Sm*SULT-OR, we created plausible models of CIDD-0072229 bound to *Sh*SULT-OR and *Sj*SULT-OR (Fig 5D). Redocking of CIDD-0072229 to these complexes returned docking scores -9.622 and -10.476 for *Sh*SULT-OR and *Sj*SULT-OR, correspondingly. These scores are similar to that of *Sm*SULT-OR which coincides with observed killing potency of the compound. The shapes of the binding sites have noticeable differences, but available space allows accommodation of the ligand in each of these enzymes. With *Sj*SULT-OR, the compound binds differently than with the other two species. In the position equivalent to Phe43 of *Sm*SULT-OR, the Leu substitution has a smaller volume. This creates a larger hydrophobic void which is efficiently occupied by the trifluoromethyl group of CIDD-0072229. It is worth noting that the shape of the compound prevents its entry into the active site through the opening formed by α6, α7 and α8 structural elements [24]. The models reveal that more global structural changes should take place for CIDD-0072229 to bind to the active site. Overall, these models are in line with the observed experimental results allowing us to rationalize the observed potency of the CIDD-0072229 across all studied species.

Schistosome killing activity achieved with compound CIDD-0072229 has shown the potential to interact with the hydrophobic pocket formed by residues Phe39, Ile42, Phe43, Leu147, Leu236 and Leu256 (S5 Fig). Indeed, the crystal structures of *Sm*SULT-OR in complex with the compounds CIDD-000204 and CIDD-000206 as well as modelling studies of other potent inhibitors **12a, 12d, 13b** from our previous study (Fig 4 from [37]) show that their benzylic moieties fit into the pocket and form extensive van der Waals contacts. The crystal structure of the *Sm*SULT-OR-CIDD-000206 complex revealed an alternative conformation (S4 Fig), where the indole moiety of CIDD-000204 binds to an opening between residues Pro134, Glu141, Tyr154 and Glu158. In this conformation, the indole ring forms a hydrogen bond with Glu141 and π−π stacking with Tyr154. Superposition of complexes with CIDD-000204 and CIDD-000206 provide a plausible route to design a compound that would combine features of both of the binding modes–modification of the central saturated ring can be used to grow molecules into both directions (S6B Fig). We enumerated a number of derivatives of CIDD-0072229, where the central saturated ring, which serves as a linker, was varied. Docking of these structures predicted 3,3-disubstituted pyrrolidine to have the highest score (S6C Fig). With this molecule, its S*-*isomer has its basic nitrogen facing the protein interior, whereas the nitrogen of the R*-*isomer is positioned toward the opening. This core was used to design compound CIDD-0149830 by linking an indole ring through a methylene linker (S6D Fig). Since CIDD-0149830 is a racemic mixture we have modelled both isomers. Docking experiment revealed strong binding for both S*-* and R*-* isomers, with docking scores -10.762 and -10.064 (lower is better), respectively. Interestingly, the S*-*isomer has its benzyl and indole moieties flipped, whereas the R-isomer has produced a pose recapitulating the designed model (S6D Fig).

## Discussion

The current drug of choice for treating human schistosomiasis, praziquantel, has yet to have a fully described mechanism of action [47–49]. What is known, however, is that OXA does not share the same mechanism of action with PZQ. Drugs that use completely different mechanisms of action are most suitable for combination therapy due to the lower probability that two completely independent mechanisms of resistance appear in a single eukaryotic parasite. Unfortunately, OXA is only effective against *S*. *mansoni*. However, our recent discovery of the gene coding for the enzyme required to activate OXA in *S*. *mansoni* (*Sm*SULT-OR) and the identification of the homologous sequences in *S*. *haematobium* and *S*. *japonicum* [25] allow us to make a very efficient rational design of derivatives. Thus, we designed OXA derivatives with the goal of developing a drug that is effective against all three major human schistosome species.

The OXA analogs described herein were designed based on an iterative structure-guided drug design approach utilizing X-ray crystallographic data obtained by Valentim *et al*. [25] to maximize favorable binding interactions, physicochemical property parameters based on Lipinski’s Rule of 5 [50], and the structure-activity relationship data from schistosomicidal efficacy of the derivatives *in vitro*. The design of the new analogs also avoided structural features or functional groups associated with known toxicities or drug development-associated hurdles. Since cost-effectiveness of a new therapy is of high importance due to the impact of the schistosomiasis endemic on developing countries and poor and rural communities, these new analogs are prepared via a short and efficient 6-step, high-yielding synthesis starting from inexpensive and readily available reagents and materials [37].

Three OXA derivatives were schistosomicidal against all three human species of *Schistosoma*. The best derivative was CIDD-0149830 which was designed as a hybrid branched structure based on the crystal structures of CIDD-0000204 and CIDD-0000206 [37] as the R-groups of the two derivatives each bound different regions in the *Sm*SULT-OR active site (S4 and S6 Figs). CIDD-0149830 killed 100% of all three human schistosome species within 7 days. Another positive property of CIDD-0149830 is its ability to kill mature female worms (Fig 4) although only 60% of females in pairs were killed. This could be attributed to the male protecting the female from the drug while en copula. There is no significant difference in killing between single mature male worms and paired male worms. *In vivo* studies should be able to answer this question. While CIDD-0066790 was only 40% schistosomicidal against *S*. *haematobium*, its R-enantiomer, CIDD-00722229 was 95% active. It is significant that we have identified two derivatives that kill *S*. *haematobium* adult worms as good as, or better than, hycanthone, a potent drug against *S*. *haematobium* but with carcinogenic properties. A recent study [51] has demonstrated excellent efficacy of OXA derivatives against *S*. *mansoni* both *in vitro* (100% killing) and *in vivo* (100% killing with a 200 mg / kg dose) and *S*. *haematobium in vitro* (75% killing activity). In addition to *S*. *mansoni* and *S*. *haematobium*, we also demonstrated 100% killing activity *by CIDD-0149830* and 90% killing activity of the R*-*enantiomer of CIDD-0066790 against *S*. *japonicum*. Hess *et al*. synthesized ruthenocenyl- and ferrocenyl-based organometallic oxaminiquine conjugates in an effort to improve ADME (absorption, distribution, metabolism, and excretion) and physicochemical properties [51]. Considering the exceptional safety, pharmacokinetic and efficacy profile of OXA itself in humans, along with the high production costs and diminishing supply of drug substance partially due to a biotransformation hydroxylation process [52, 53], our approach focused on developing a novel small molecule that possessed a similar physicochemical profile to OXA, but was not dependent on starting from the parent OXA drug itself. Thus, we developed a structure-based drug design strategy to identify novel compounds that had efficacious broad-range anti-schistosomal activity, favorable “drug-like” ADME and physicochemical properties, and ultimately provided an opportunity for a much more simplified and efficient synthesis approach, amenable to a larger-scale synthesis process. Since CIDD-0066790 and CIDD-0149830 have been reluctant to co-crystallize or soak into crystals for any of the sulfotransferases, the precise reason for their efficacy is heretofore unknown.

In order to provide further insight into the binding of CIDD-0072229 (the R-enantiomer of CIDD-0066790) we performed a docking analysis of CIDD-0072229 with *Sm*SULT-OR, *Sh*SULT-OR and *Sj*SULT-OR. The generated models of CIDD-0072229 bound to *Sm*SULT-OR, *Sh*SULT-OR and *Sj*SULT-OR were plausible. Flexibility of the CIDD-0072229, as well as inherent plasticity of the schistosomal sulfotransferase binding sites, accommodated the ligand in nearly identical conformations across the three SULT-OR models. By analogy to OXA binding in *Sm*SULT-OR and *Sh*SULT-OR crystal structures, these models allow us to rationalize the observed potency of the CIDD-0072229 across all schistosome species studied. We hypothesize that the difference in activation and thus killing is due to the ability of the derivative to fit in the binding pocket of each sulfotransferase (*Sm*SULT-OR, *Sh*SULT-OR, *Sj*SULT-OR) and to be efficiently sulfated [24].

Our very best derivative for pan species killing of schistosomes is CIDD-0149830. It kills 100% of *S*. *mansoni*, *S*. *haematobium* and *S*. *japonicum* within 7 days. To date, we have not been successful in soaking CIDD-0149830 into SULT-OR crystals of any schistosome species.

The concern about drug resistance evolving against praziquantel is a significant danger with serious health implications for control. History has taught that monotherapy is not a good control strategy especially against hyperendemic diseases that are being primarily controlled by drug therapy (e.g. malaria, HIV). In light of this concern, there have been efforts to develop new drugs to be used in combination with praziquantel [27, 54–56].

A directed target-based approach for drug discovery is powerful as demonstrated by the present study and others [47, 48].

### Conclusion

Using data gleaned from the crystallographic studies and in collaboration with a medicinal chemist and X-ray crystallography laboratory, OXA derivatives were produced, and screened *in vitro* against all three major species of schistosomes. CIDD-0149830, CIDD-0066790 and its enantiomer CIDD-00722229 were identified as the most promising pharmaceutical candidates. These derivatives showed significant worm killing of all three worm species of schistosomes. All of the derivatives were designed based on OXA and likely are themselves, prodrugs which would require SULT-OR activity to become activated. Initial *in vitro* tests indicate that SULT-OR activity is responsible for CIDD-0066790 activation.

While the current drug praziquantel is effective and extraordinarily economical, there is no second drug to turn to when mass chemotherapy causes enough selective pressure for resistance to sweep through the parasite population. The recently elucidated mechanism of action for an older drug, OXA, has created an exploitable opening for directed drug development. The distinct difference in the mechanism of action in the drug of choice, PZQ, to OXA and its derivatives allows for a viable combination therapy to combat emerging resistance. Next, we will test the best derivatives in an *in vivo* model, demonstrate that the OXA derivatives can kill PZQ resistant worms and determine the combination efficacy of OXA-derivative and PZQ.

## Supporting information

S1 Fig Effect of CIDD-149830 on *S*. *mansoni* worm pairs, mature males and mature females. OXA derivatives were tested against adult male, female and worm pairs of *S*. *mansoni in vitro*. The OXA derivatives were tested as described in Figure legend 3.
(TIF)

S2 Fig SmSULT-OR expression in Male and Female Adult Worms by qRT-PCR. Transcripts of the SmSULT-OR gene from male and female *S*. *mansoni* were evaluated by quantitative reverse transcriptase PCR. GAPDH was used as an internal reference and relative quantities were determined by the ΔΔCt method. Results represent three replicates.
(TIF)

S3 Fig Dose-dependent response of *S*. *mansoni* to CIDD-00149830. Male *S*. *manson*i worms were treated with 143 μm, 71.5 μm or 35.75 μM and observed for 14 days.
(TIF)

S4 Fig Chemical structures (top panel) of CIDD-000204 (left) and CIDD-000206 (right) (37). Crystal structures (lower panel) of CIDD-000204 (left, PDB entry 6BDS) and CIDD-000206 (right, PDB entry 6BDR) complexed with *Sm*SULT-OR. The position of bound OXA in *Sm*SULT-OR (from PDB entry 5BYK) is shown overlaid for comparison. Figure was generated using PyMOL (Schrödinger, LLC).
(TIF)

S5 Fig Interaction diagram of CIDD-0072229 bound to *Sm*SULT-OR. The hydrophobic moiety of the compound forms extensive interactions with apolar residues of the sulfotransferase.
(TIF)

S6 Fig Design of CIDD-0149830. **A** Superposition of the CIDD-0072229 derived from docking over the X-ray structure of CIDD-000204 (PDB entry 6BDS) shows efficient overlap of aromatic moieties of both compounds, whereas the saturated ring can be varied. **B** Superposition of CIDD-000204 and CIDD-000206 provides a rational basis to combine two ligands into one molecule. **C** Best scored derivative of CIDD-0072229 has its nitrogen facing an opening formed by α6, α7 and α8 helices. **D** Superposition of the docking pose of CIDD-0149830 over CIDD-000204 and CIDD-000206 shows extensive overlap between these compounds. To improve visualization, the names of compounds are shortened to the last three digits and α6 helix is removed from the cartoon representation.
(TIF)

S1 Table Synthesis of CIDD Compounds. (DOCX)

## References

[1] Gryseels B, Polman K, Clerinx J, Kestens L. Human schistosomiasis. Lancet. 2006;368(9541):1106–18. Epub 2006/09/26. 10.1016/S0140-6736(06)69440-3 16997665

[2] Steinmann P, Keiser J, Bos R, Tanner M, Utzinger J. Schistosomiasis and water resources development: systematic review, meta-analysis, and estimates of people at risk. The Lancet Infectious diseases. 2006;6(7):411–25. Epub 2006/06/23. 10.1016/S1473-3099(06)70521-7 16790382

[3] WHO. Fact Sheet: Schistosomiasis. 2016; Available from:.

[4] Chitsulo L, Engels D, Montresor A, Savioli L. The global status of schistosomiasis and its control. Acta tropica. 2000;77(1):41–51. Epub 2000/09/21. 10.1016/s0001-706x(00)00122-4 10996119 PMC5633072

[5] Committee WE. Prevention and control of schistosomiasis and soil-transmitted helminthiasis. World Health Organization technical report series. 2002;912:i-vi, 1–57, back cover. Epub 2003/02/21. 12592987

[6] van der Werf MJ, de Vlas SJ, Brooker S, Looman CW, Nagelkerke NJ, Habbema JD, et al Quantification of clinical morbidity associated with schistosome infection in sub-Saharan Africa. Acta tropica. 2003;86(2–3):125–39. Epub 2003/05/15. 10.1016/s0001-706x(03)00029-9 12745133

[7] Collaborators GDaH. Global, regional, and national disability-adjusted life-years (DALYs) for 315 diseases and injuries and healthy life expectancy (HALE), 1990–2015: a systematic analysis for the Global Burden of Disease Study 2015. Lancet. 2016;388(10053):1603–58. Epub 2016/10/14. 10.1016/S0140-6736(16)31460-X 27733283 PMC5388857

[8] King CH. Lifting the burden of schistosomiasis—defining elements of infection-associated disease and the benefits of antiparasite treatment. The Journal of infectious diseases. 2007;196(5):653–5. Epub 2007/08/04. 10.1086/520522 17674304

[9] King CH. Schistosomiasis Japonica: The DALYs Recaptured. PLoS neglected tropical diseases. 2008;2(3):e203 Epub 2008/03/06. 10.1371/journal.pntd.0000203 18320017 PMC2254200

[10] King CH. Parasites and poverty: the case of schistosomiasis. Acta tropica. 2010;113(2):95–104. Epub 2009/12/08. 10.1016/j.actatropica.2009.11.012 19962954 PMC2812649

[11] Fenwick A, Webster JP, Bosque-Oliva E, Blair L, Fleming FM, Zhang Y, et al The Schistosomiasis Control Initiative (SCI): rationale, development and implementation from 2002–2008. Parasitology. 2009;136(13):1719–30. Epub 2009/07/28. 10.1017/S0031182009990400 19631008

[12] Vale N, Gouveia MJ, Rinaldi G, Brindley PJ, Gartner F, Correia da Costa JM. Praziquantel for Schistosomiasis: Single-Drug Metabolism Revisited, Mode of Action, and Resistance. Antimicrobial agents and chemotherapy. 2017;61(5). Epub 2017/03/08. 10.1128/AAC.02582-16 PMC5404606 28264841

[13] WHO. Zanzibar: gearing up to eliminate schistosomiasis. 2012; Available from: http://www.who.int/neglected_diseases/schistosomiasis_zanzibar_2012/en.

[14] Cioli D, Pica-Mattoccia L, Archer S. Antischistosomal drugs: past, present… and future? Pharmacology & therapeutics. 1995;68(1):35–85. Epub 1995/01/01. 8604437 10.1016/0163-7258(95)00026-7

[15] Katz N, Coelho PM. Clinical therapy of schistosomiasis mansoni: the Brazilian contribution. Acta tropica. 2008;108(2–3):72–8. Epub 2008/07/12. 10.1016/j.actatropica.2008.05.006 18617155

[16] Hagan P, Appleton CC, Coles GC, Kusel JR, Tchuem-Tchuente LA. Schistosomiasis control: keep taking the tablets. Trends in parasitology. 2004;20(2):92–7. Epub 2004/01/30. 10.1016/j.pt.2003.11.010 14747023

[17] Archer S, Yarinsky A. Recent developments in the chemotherapy of schistosomiasis. Progress in drug research Fortschritte der Arzneimittelforschung Progres des recherches pharmaceutiques. 1972;16:11–66. Epub 1972/01/01. 10.1007/978-3-0348-7081-8_1 4633870

[18] Haese WH, Bueding E. Long-term hepatocellular effects of hycanthone and of two other anti-Schistosomal drugs in mice infected with Schistosoma mansoni. The Journal of pharmacology and experimental therapeutics. 1976;197(3):703–13. Epub 1976/06/01. 180276

[19] Hartman PE, Hulbert PB. Genetic activity spectra of some antischistosomal compounds, with particular emphasis on thioxanthenones and benzothiopyranoindazoles. Journal of toxicology and environmental health. 1975;1(2):243–70. Epub 1975/11/01. 10.1080/15287397509529325 1107580

[20] Pica-Mattoccia L, Dias LC, Moroni R, Cioli D. Schistosoma mansoni: genetic complementation analysis shows that two independent hycanthone/oxamniquine-resistant strains are mutated in the same gene. Experimental parasitology. 1993;77(4):445–9. Epub 1993/12/01. 10.1006/expr.1993.1104 8253157

[21] Gentile R, Oliveira G. Brazilian studies on the genetics of Schistosoma mansoni. Acta tropica. 2008;108(2–3):175–8. Epub 2008/10/04. 10.1016/j.actatropica.2008.09.003 18831955 PMC2615404

[22] Katz N, Dias EP, Araujo N, Souza CP. Estudo de uma cepa humana de Schistosoma mansoni resistente a agentes esquistossomicidas. Revista da Sociedade Brasileira de Medicina Tropical. 1973;7(6):381–7.

[23] Rogers SH, Bueding E. Hycanthone resistance: development in Schistosoma mansoni. Science. 1971;172(3987):1057–8. Epub 1971/06/04. 10.1126/science.172.3987.1057 5103321

[24] Taylor AB, Roberts KM, Cao X, Clark NE, Holloway SP, Donati E, et al Structural and enzymatic insights into species-specific resistance to schistosome parasite drug therapy. The Journal of biological chemistry. 2017;292(27):11154–64. Epub 2017/05/26. 10.1074/jbc.M116.766527 28536265 PMC5500785

[25] Valentim CL, Cioli D, Chevalier FD, Cao X, Taylor AB, Holloway SP, et al Genetic and molecular basis of drug resistance and species-specific drug action in schistosome parasites. Science. 2013;342(6164):1385–9. Epub 2013/11/23. 10.1126/science.1243106 24263136 PMC4136436

[26] Cioli D, Pica-Mattoccia L, Archer S. Resistance of schistosomes to hycanthone and oxamniquine. Memorias do Instituto Oswaldo Cruz. 1989;84 Suppl 1:38–45. Epub 1989/10/01. 2638729 10.1590/s0074-02761989000500005

[27] da Silva VBR, Campos B, de Oliveira JF, Decout JL, do Carmo Alves de Lima M. Medicinal chemistry of antischistosomal drugs: Praziquantel and oxamniquine. Bioorganic & medicinal chemistry. 2017;25(13):3259–77. Epub 2017/05/13. 28495384 10.1016/j.bmc.2017.04.031

[28] Guzman MA, Rugel A, Tarpley RS, Cao X, McHardy SF, LoVerde PT, et al Molecular Basis for Hycanthone Drug Action in Schistosome Parasites. Molecular and biochemical parasitology. 2020; 236:111257 10.1016/j.molbiopara.2020.111257 32027942 PMC13162085

[29] Pica-Mattoccia L, Cioli D, Archer S. Binding of oxamniquine to the DNA of schistosomes. Transactions of the Royal Society of Tropical Medicine and Hygiene. 1989;83(3):373–6. Epub 1989/05/01. 10.1016/0035-9203(89)90508-7 2617584

[30] LoVerde PT, Niles EG, Osman A, Wu W. Schistosoma mansoni male–female interactions. Canadian Journal of Zoology. 2004;82(2):357–74.

[31] Alonso D, Munoz J, Gascon J, Valls ME, Corachan M. Failure of standard treatment with praziquantel in two returned travelers with Schistosoma haematobium infection. The American journal of tropical medicine and hygiene. 2006;74(2):342–4. Epub 2006/02/14. 16474094

[32] Gryseels B, Stelma FF, Talla I, van Dam GJ, Polman K, Sow S, et al Epidemiology, immunology and chemotherapy of Schistosoma mansoni infections in a recently exposed community in Senegal. Tropical and geographical medicine. 1994;46(4 Spec No):209–19. Epub 1994/01/01. 7825223

[33] Ismail M, Botros S, Metwally A, William S, Farghally A, Tao LF, et al Resistance to praziquantel: direct evidence from Schistosoma mansoni isolated from Egyptian villagers. The American journal of tropical medicine and hygiene. 1999;60(6):932–5. Epub 1999/07/14. 10.4269/ajtmh.1999.60.932 10403323

[34] Couto FF, Coelho PM, Araujo N, Kusel JR, Katz N, Jannotti-Passos LK, et al Schistosoma mansoni: a method for inducing resistance to praziquantel using infected Biomphalaria glabrata snails. Memorias do Instituto Oswaldo Cruz. 2011;106(2):153–7. Epub 2011/05/04. 10.1590/s0074-02762011000200006 21537673

[35] Fallon PG, Doenhoff MJ. Drug-resistant schistosomiasis: resistance to praziquantel and oxamniquine induced in Schistosoma mansoni in mice is drug specific. The American journal of tropical medicine and hygiene. 1994;51(1):83–8. Epub 1994/07/01. 10.4269/ajtmh.1994.51.83 8059919

[36] Duvall RH, DeWitt WB. An improved perfusion technique for recovering adult schistosomes from laboratory animals. The American journal of tropical medicine and hygiene. 1967;16(4):483–6. Epub 1967/07/01. 10.4269/ajtmh.1967.16.483 4952149

[37] Rugel A, Tarpley RS, Lopez A, Menard T, Guzman MA, Taylor AB, et al Design, Synthesis, and Characterization of Novel Small Molecules as Broad Range Antischistosomal Agents. ACS medicinal chemistry letters. 2018;9(10):967–73. Epub 2018/10/23. 10.1021/acsmedchemlett.8b00257 30344901 PMC6187409

[38] Schrödinger L. Small-Molecule Drug Discovery Suite. 2018–1 ed. New York, NY2018.

[39] Abraham MJ, Murtola T, Schulz R, Páll S, Smith JC, Hess B, et al GROMACS: High performance molecular simulations through multi-level parallelism from laptops to supercomputers. SoftwareX. 2015;1–2:19–25.

[40] Humphrey W, Dalke A, Schulten K. VMD: visual molecular dynamics. Journal of molecular graphics. 1996;14(1):33–8, 27–8. Epub 1996/02/01. 10.1016/0263-7855(96)00018-5 8744570

[41] Pettersen EF, Goddard TD, Huang CC, Couch GS, Greenblatt DM, Meng EC, et al UCSF Chimera—a visualization system for exploratory research and analysis. Journal of computational chemistry. 2004;25(13):1605–12. Epub 2004/07/21. 10.1002/jcc.20084 15264254

[42] Rugel A, Guzman MA, Taylor AB, Chevalier FD, Tarpley RS, McHardy SF, Cao X, Holloway SP, Anderson TJC, Hart PJ, LoVerde PT. Why does oxamniquine kill *Schistosoma mansoni* and not *S*. *haematobium* and *S*. *japonicum*? Internatl J Parasitol Drugs and Drug Resistance. 2020;13:8–15. 10.1016/j.ijpddr.2020.04.001 PMC7167500 32315953

[43] Friesner RA, Murphy RB, Repasky MP, Frye LL, Greenwood JR, Halgren TA, et al Extra precision glide: docking and scoring incorporating a model of hydrophobic enclosure for protein-ligand complexes. Journal of medicinal chemistry. 2006;49(21):6177–96. Epub 2006/10/13. 10.1021/jm051256o 17034125

[44] Therneau TM. A Package for Survival Analysis in S. 2.38 ed2015.

[45] Taylor AB, Pica-Mattoccia L, Polcaro CM, Donati E, Cao X, Basso A, et al Structural and Functional Characterization of the Enantiomers of the Antischistosomal Drug Oxamniquine. PLoS neglected tropical diseases. 2015;9(10):e0004132 Epub 2015/10/21. 10.1371/journal.pntd.0004132 26485649 PMC4618941

[46] Watts KS, Dalal P, Murphy RB, Sherman W, Friesner RA, Shelley JC. ConfGen: a conformational search method for efficient generation of bioactive conformers. Journal of chemical information and modeling. 2010;50(4):534–46. Epub 2010/04/09. 10.1021/ci100015j 20373803

[47] Caffrey CR. Chemotherapy of schistosomiasis: present and future. Current opinion in chemical biology. 2007;11(4):433–9. Epub 2007/07/27. 10.1016/j.cbpa.2007.05.031 17652008

[48] Doenhoff MJ, Cioli D, Utzinger J. Praziquantel: mechanisms of action, resistance and new derivatives for schistosomiasis. Current opinion in infectious diseases. 2008;21(6):659–67. Epub 2008/11/04. 10.1097/QCO.0b013e328318978f 18978535

[49] Thomas CM, Timson DJ. The Mechanism of Action of Praziquantel: Six Hypotheses. Current topics in medicinal chemistry. 2018;18(18):1575–84. Epub 2018/10/30. 10.2174/1568026618666181029143214 30370849

[50] Lipinski CA. Lead- and drug-like compounds: the rule-of-five revolution. Drug discovery today Technologies. 2004;1(4):337–41. Epub 2004/12/01. 10.1016/j.ddtec.2004.11.007 24981612

[51] Hess J, Panic G, Patra M, Mastrobuoni L, Spingler B, Roy S, et al Ferrocenyl, Ruthenocenyl, and Benzyl Oxamniquine Derivatives with Cross-Species Activity against Schistosoma mansoni and Schistosoma haematobium. ACS infectious diseases. 2017;3(9):645–52. Epub 2017/07/08. 10.1021/acsinfecdis.7b00054 28686009

[52] Adlercreutz P, Straathof AJJ. Applied biocatalysis. 2nd ed Amsterdam: Harwood Academic Publishers; 2000 xvi, 443 p., 9 p. of plates p.

[53] Boehm D, Pryce DJ. Schistosoma haematobium. The New England journal of medicine. 2001;344(15):1170. Epub 2001/04/17. 10.1056/NEJM200104123441515 11302147

[54] Cioli D, Valle C, Angelucci F, Miele AE. Will new antischistosomal drugs finally emerge? Trends in parasitology. 2008;24(9):379–82. Epub 2008/08/05. 10.1016/j.pt.2008.05.006 18675590

[55] da Rocha Pitta MG, da Rocha Pitta MG, de Melo Rego MJ, Galdino SL. The evolution of drugs on schistosoma treatment: looking to the past to improve the future. Mini reviews in medicinal chemistry. 2013;13(4):493–508. Epub 2013/02/05. 10.2174/1389557511313040003 23373654

[56] Geary TG, Sakanari JA, Caffrey CR. Anthelmintic drug discovery: into the future. The Journal of parasitology. 2015;101(2):125–33. Epub 2015/01/15. 10.1645/14-703.1 25584662

