# Supplementary figures and images for: An iterative process produces oxamniquine derivatives that kill the major species of schistosomes infecting humans
Source: PLoS Negl Trop Dis. 2020 Aug 18;14(8):e0008517. doi: 10.1371/journal.pntd.0008517 (PMC7454593; doi:10.1371/journal.pntd.0008517)

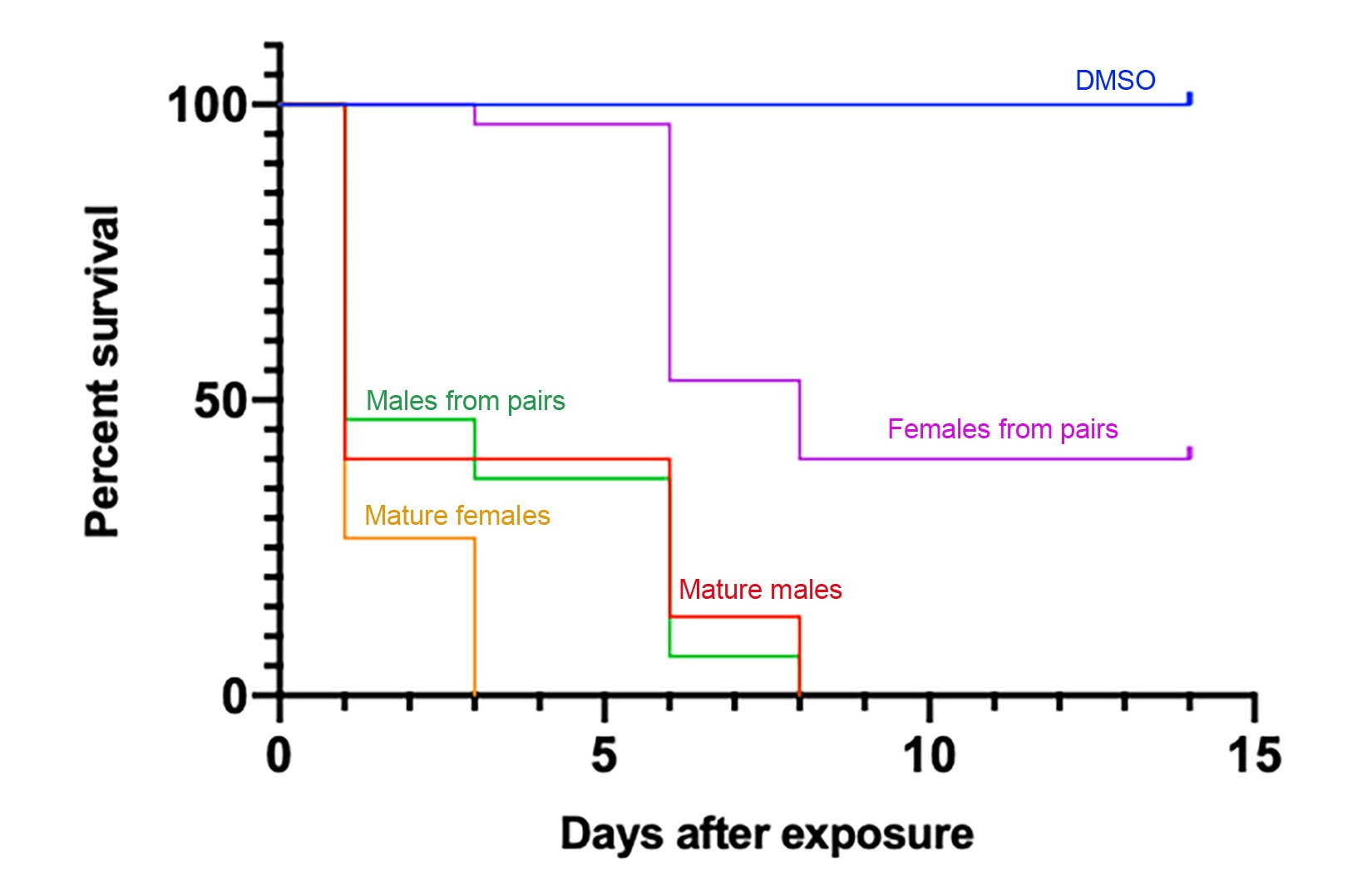

Supplement: S1 Fig — OXA derivatives were tested against adult male, female and worm pairs of S. mansoni in vitro. The OXA derivatives were tested as described in Figure legend 3. (TIF) [file pntd.0008517.s001.tif]

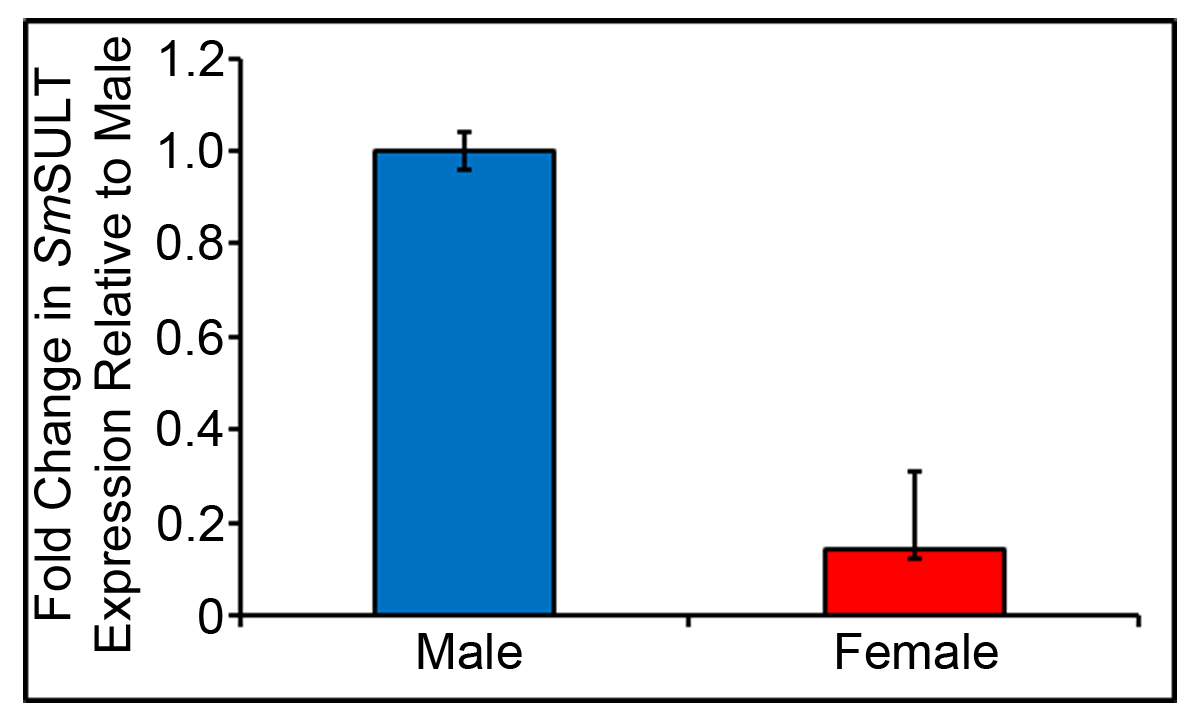

Supplement: S2 Fig — Transcripts of the SmSULT-OR gene from male and female S. mansoni were evaluated by quantitative reverse transcriptase PCR. GAPDH was used as an internal reference and relative quantities were determined by the ΔΔCt method. Results represent three replicates. (TIF) [file pntd.0008517.s002.tif]

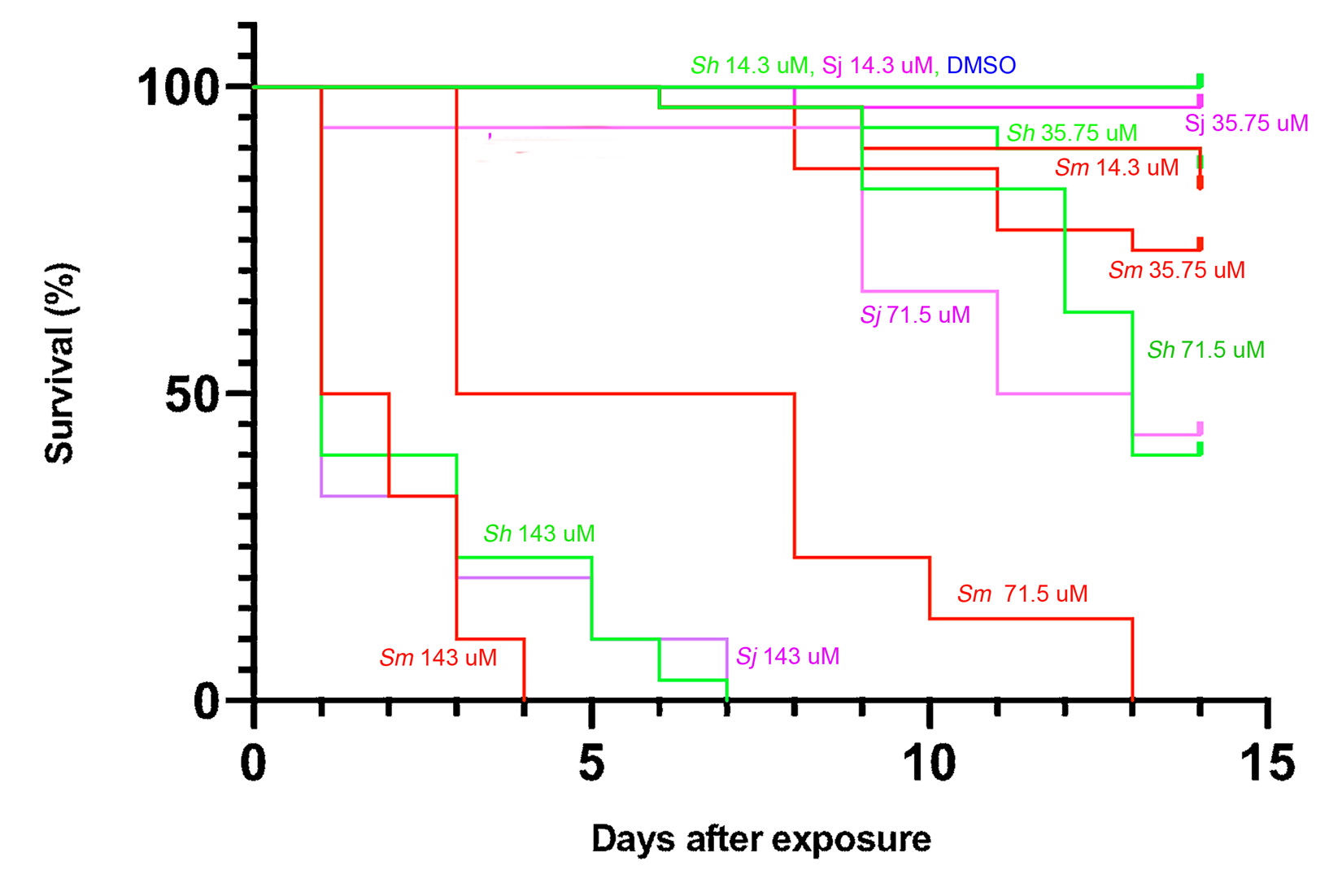

Supplement: S3 Fig — Male S. mansoni worms were treated with 143 μm, 71.5 μm or 35.75 μM and observed for 14 days. (TIF) [file pntd.0008517.s003.tif]

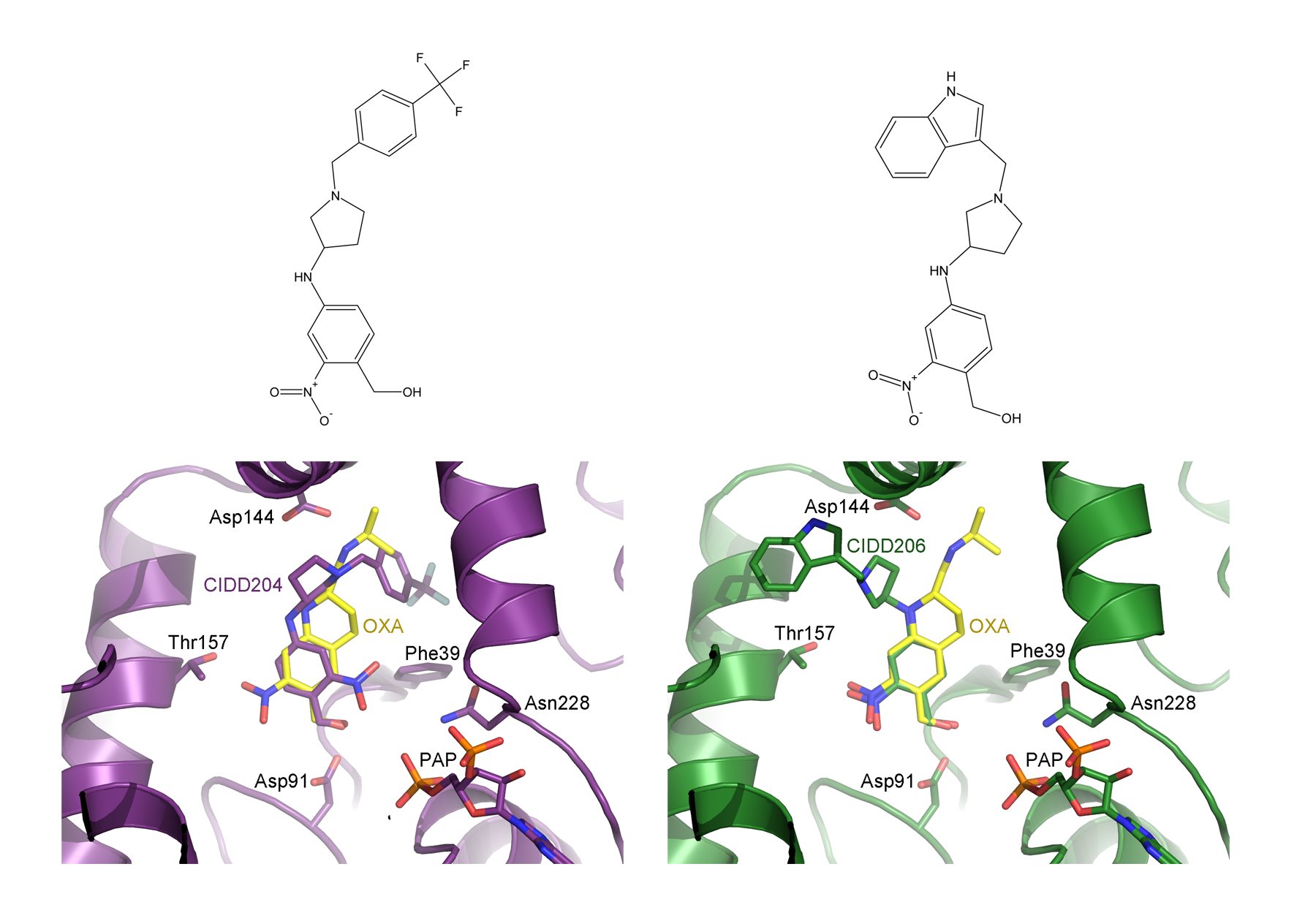

Supplement: S4 Fig — Chemical structures (top panel) of CIDD-000204 (left) and CIDD-000206 (right) (37). Crystal structures (lower panel) of CIDD-000204 (left, PDB entry 6BDS) and CIDD-000206 (right, PDB entry 6BDR) complexed with SmSULT-OR. The position of bound OXA in SmSULT-OR (from PDB entry 5BYK) is shown overlaid for comparison. Figure was generated using PyMOL (Schrödinger, LLC). (TIF) [file pntd.0008517.s004.tif]

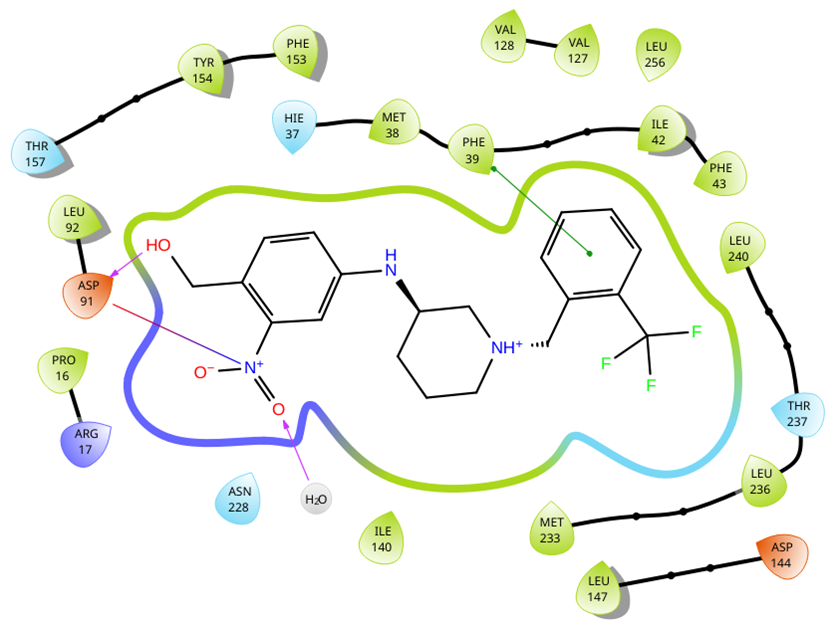

Supplement: S5 Fig — The hydrophobic moiety of the compound forms extensive interactions with apolar residues of the sulfotransferase. (TIF) [file pntd.0008517.s005.tif]

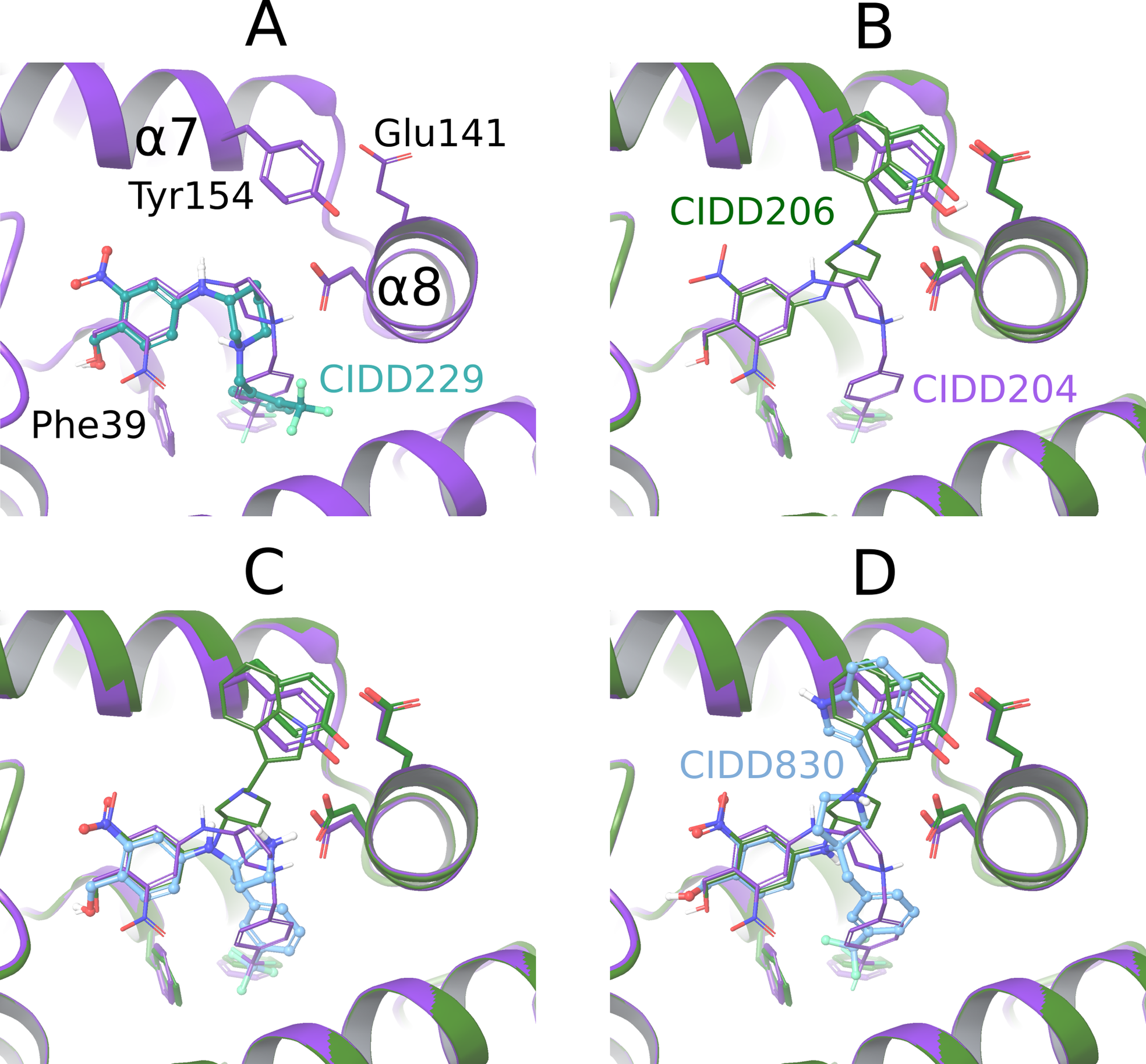

Supplement: S6 Fig — A Superposition of the CIDD-0072229 derived from docking over the X-ray structure of CIDD-000204 (PDB entry 6BDS) shows efficient overlap of aromatic moieties of both compounds, whereas the saturated ring can be varied. B Superposition of CIDD-000204 and CIDD-000206 provides a rational basis to combine two ligands into one molecule. C Best scored derivative of CIDD-0072229 has its nitrogen facing an opening formed by α6, α7 and α8 helices. D Superposition of the docking pose of CIDD-0149830 over CIDD-000204 and CIDD-000206 shows extensive overlap between these compounds. To improve visualization, the names of compounds are shortened to the last three digits and α6 helix is removed from the cartoon representation. (TIF) [file pntd.0008517.s006.tif]
