# Supplementary material for: An iterative process produces oxamniquine derivatives that kill the major species of schistosomes infecting humans
Source: PLoS Negl Trop Dis. 2020 Aug 18;14(8):e0008517. doi: 10.1371/journal.pntd.0008517 (PMC7454593; doi:10.1371/journal.pntd.0008517)
Supplement: S1 Table — (DOCX) [file pntd.0008517.s007.docx]

**Experimental Section**

**Synthesis of CIDD Compounds**

**Synthesis of CIDD-0072398, CIDD-0072298, CIDD-0072300, CIDD-0072228 and CIDD-0072229**

The compounds CIDD-0072398, CIDD-0072298, CIDD-0072300, CIDD-0072228 and CIDD-0072229 were prepared according to the synthesis schemes and experimental procedures described by A. Rugel, et al, *ACS Med. Chem. Letters,* **2018***, 9,* 967-973. Analytical data for each compound is provided below, along with chiral HPLC data for CIDD-0072228 and CIDD-0072229.

**CIDD-0072398**

^1^H NMR (400 MHz, CD_3_OD) δ 7.68 (t, J = 8.0 Hz, 1H), 7.49 (d, J = 7.7 Hz, 1H), 7.44 (d, J = 8.5 Hz, 2H), 7.19 (d, J = 2.5 Hz, 1H), 6.89 (dd, J = 8.5, 2.5 Hz, 1H), 4.75 (s, 2H), 4.04 (m, 1H), 3.81 (ABq, ΔV_AB_ = 7.7 Hz, *J_AB_* = 14.0 Hz , 2H), 2.98 (dd, J = 9.6, 6.9 Hz, 1H), 2.80 (td, J = 8.5, 6.1 Hz, 1H), 2.64 (m, 1H), 2.56 (dd, J = 9.7, 4.5 Hz, 1H), 2.35 (m, 1H), 1.71 (m, 1H). ^13^C NMR (400 MHz, cd3od) δ 160.73 (d, *J* = 246 Hz), 148.57, 148.12, 132.28, 138.24, 129.88, 129.60, 129.44, 123.69, 120.62 (q, *J* = 15.2 Hz), 117.26, 112.20 (dq, *J* = 24.8, 4.8 Hz), 108.68, 60.44, 59.70, 52.39, 51.81, 51.46, 31.55. ESI-MS (m/z): 414.2 [M +1]. HRMS (EI): m/z calcd for C_19_H_19_F_4_N_3_O_3_ [M+1]: 414.1435; found, 414.1431.

**CIDD-0072298**

^1^H NMR (400 MHz, CDCl_3_) δ 7.87 (dd, *J* = 8.5, 5.8 Hz, 1H), 7.45 (d, *J* = 8.7 Hz, 2H), 7.38 (td, *J* = 8.5, 2.7 Hz, 1H), 7.21 (d, *J* = 2.4 Hz, 1H), 6.91 (dd, *J* = 8.5, 2.4 Hz, 1H), 4.75 (s, 2H), 4.08 (m, 1H), 3.90 (ABq, ΔV_AB_ = 9.8 Hz, *J_AB_* = 14.0 Hz , 2H), 3.02 (dd, *J* = 9.8, 6.7 Hz, 1H), 2.89 (m, 1H), 2.71 (m, 1H), 2.65 (dd, *J* = 9.9, 4.4 Hz, 1H), 2.38 (tdd, *J* = 14.4, 8.4, 6.3 Hz, 1H), 1.78 (m, 1H).^13^C NMR (400 MHz, cd3od) δ 162.47 (d, *J* = 246 Hz) 150.00, 149.61, 135.3, 134.21, 131.31, 125.03, 120.06, 119.86, 118.71, 114.03 (dq, *J* = 25.0, 6.0 Hz), 108.68, 61.89, 61.33, 56.13, 56.10, 53.99, 53.39, 33.05. ESI-MS (m/z): 414.1 [M +1]. HRMS (EI): m/z calcd for C_19_H_19_F_4_N_3_O_3_ [M+1]: 414.1435; found, 414.1436.


**CIDD-0072300**
^1^H NMR (400 MHz, CD_3_OD) δ 7.53 (d, *J* = 1.9 Hz, 1H), 7.44 (t, *J* = 8.8 Hz, 2H), 7.27 (dd, *J* = 8.2, 2.0 Hz, 1H), 7.18 (d, *J* = 2.5 Hz, 1H), 6.88 (dd, *J* = 8.5, 2.5 Hz, 1H), 4.75 (s, 2H), 4.02 (m, 1H), 3.63 (ABq, ΔV_AB_ = 10.5 Hz, *J_AB_* = 12.0 Hz , 2H), 2.91 (dd, *J* = 9.7, 6.8 Hz, 1H), 2.74 (td, *J* = 8.5, 6.0 Hz, 1H), 2.57 (m, 1H), 2.50 (dd, *J* = 9.7, 4.4 Hz, 1H), 2.34 (dtd, *J* = 13.7, 8.1, 5.9 Hz, 1H), 1.70 (m, 1H). ^13^C NMR (400 MHz, cd3od) δ 148.56, 148.13, 139.23, 131.78, 130.59, 130.01, 129.88, 128.44, 123.68, 117.26, 107.24, 60.45, 59.73, 58.45, 52.41, 51.81, 31.52. ESI-MS (m/z): 396.1 [M +1]. HRMS (EI): m/z calcd for C_18_H_19_Cl_2_N_3_O_3_ [M+1]: 396.0876; found, 396.0870.

**CIDD-0072229 (R)**

^1^H NMR (400 MHz, CD_3_OD) δ 7.86 (d, *J* = 7.5 Hz, 1H), 7.64 (d, *J* = 7.9 Hz, 1H), 7.59 (t, *J* = 7.6 Hz, 1H), 7.40 (dd, *J* = 8.0, 4.2 Hz, 2H), 7.17 (d, *J* = 2.4 Hz, 1H), 6.87 (dd, *J* = 8.5, 2.4 Hz, 1H), 4.73 (s, 2H), 3.68 (ABq, *ΔV_AB_*  = 22.3 Hz, *J_AB_*=14.4 Hz, 2H), 3.54 (m, 1H), 2.87 (d, *J* = 10.5 Hz, 1H), 2.66 (m, 1H), 2.28 (m, 1H), 2.06 (m, 1H), 1.93 (m, 1H), 1.79 (m, 1H), 1.69 (m, 1H), 1.43 (m, 1H). ^13^C NMR (400 MHz, cdcl3) δ 148.62, 147.75, 137.56, 131.77, 130.26, 129.92, 128.15 (q, *J* = 29.6 Hz), 126.83, 125.99, 125.38 (q, *J* = 6.0 Hz), 123.36, 117.21, 106.90, 60.45, 58.13, 58.11, 53.64, 48.95, 29.24, 23.13. ESI-MS (m/z): 410.2 [M +1]. HRMS (EI): m/z calcd for C_20_H_22_F_3_N_3_O_3_ [M+1]: 410.1686; found, 410.1681.


**CIDD-0072228 (S)**

^1^H NMR (400 MHz, CD_3_OD) δ 7.86 (d, *J* = 7.5 Hz, 1H), 7.64 (d, *J* = 8.0 Hz, 1H), 7.59 (t, *J* = 7.7 Hz, 1H), 7.40 (dd, *J* = 8.0, 4.2 Hz, 2H), 7.17 (d, *J* = 2.4 Hz, 1H), 6.87 (dd, *J* = 8.5, 2.5 Hz, 1H), 4.73 (s, 2H), 3.68 (ABq, *ΔV_AB_* = 22.3 Hz, *J_AB_*=14.4 Hz, 2H), 3.54 (m, 1H), 2.87 (d, *J* = 13.4 Hz, 1H), 2.66 (m, 1H), 2.28 (m, 1H), 2.05 (m, 1H), 1.93 (m, 1H), 1.79 (m, 1H), 1.69 (m, 1H), 1.43 (m, 1H). ^13^C NMR (400 MHz, cdcl3) δ 152.57, 151.69, 141.50, 135.70, 134.21, 133.86, 132.10 (q, *J* = 29.2 Hz), 130.76, 129.93, 129.31 (q, *J* = 6.0 Hz), 127.28, 121.13, 110.84, 64.39, 62.07, 61.96, 57.58, 52.90, 33.26, 27.09. ESI-MS (m/z): 410.2 [M +1]. HRMS (EI): m/z calcd for C_20_H_22_F_3_N_3_O_3_ [M+1]: 410.1686; found, 410.1688.

**Chiral HPLC Analysis of CIDD-0072228 and CIDD-007229**

Chiral HPLC traces for confirmation of enantiomeric purity of CIDD-0072228 and CIDD-0072229. *Conditions:* Chiralpak AY-H (4.5 mm x 250 mm), detection at λ 254 nm, isocratic elution: 0.05% diethyl amine, 4.95% isopropanol, 95% hexanes, flow rate: 1.0 mL/min. Chiral HPLC traces for the racemic material and both CIDD-72228 and CIDD-72229 are below, confirming enantiomeric excess of 95% ee.


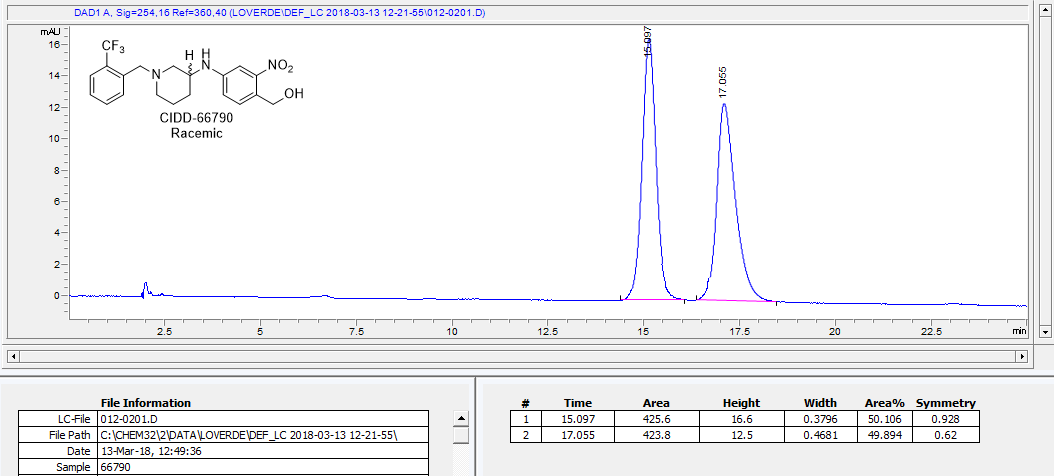


**Figure S1. Chiral HPLC trace for racemic compound, CIDD-0066790**


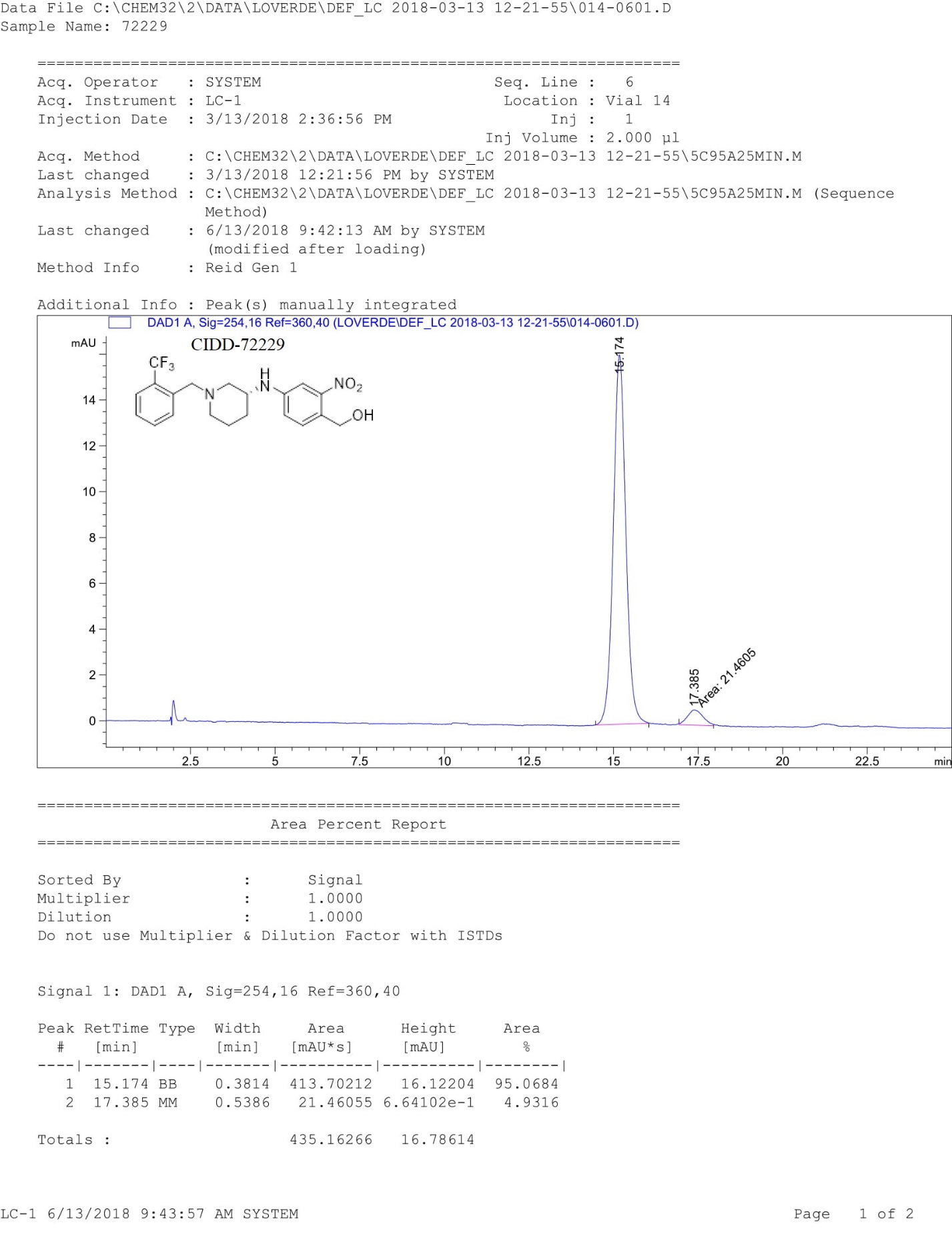


**Figure S2. Chiral HPLC trace for CIDD-0072229**


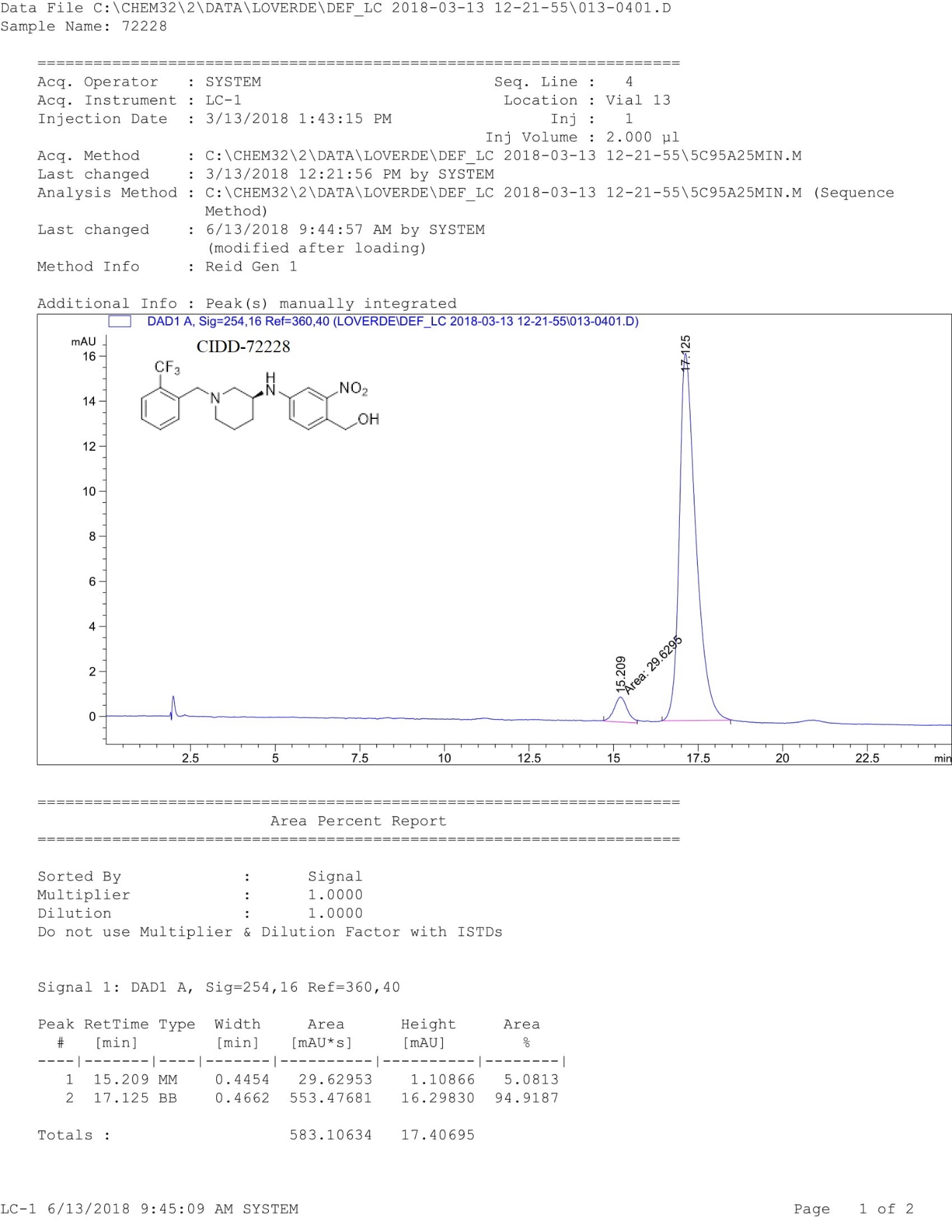


**Figure S3. Chiral HPLC trace for CIDD-0072228**

**Synthesis of CIDD-0149830**

The compound CIDD-0149830 was prepared according to the synthesis highlighted in scheme S1 and experimental procedures described below.

**Scheme S1. Synthesis of CIDD-0149830**

**General procedures.** All operations were carried out at room or ambient temperature, that is, in the range of 18-25^o^C; evaporation of solvent was carried out using a rotary evaporator under reduced pressure with a bath of up to 50^o^C; reactions were monitored by thin layer chromatography (tlc) and reaction times are given for illustration only. Unless otherwise indicated all reactions were conducted in standard commercially available glassware using standard synthetic chemistry methods and setup. All air- and moisture-sensitive reactions were performed under nitrogen atmosphere with dried solvents and glassware under anhydrous conditions. Starting materials and reagents were commercial compounds of the highest purity available and were used without purification (See list of specific reagents below). Solvents used for reactions were indicated as of commercial dry or extra-dry or analytical grade. Analytical thin layer chromatography was performed on aluminium plates coated with Merck Kieselgel 60F254 and visualized by UV irradiation (254 nm) or by staining with a solution of potassium permanganate. Flash column chromatography was performed on Biotage Isolera One 2.2 using commercial columns that were pre-packed with Merck Kieselgel 60 (230– 400 mesh) silica gel. Final compounds for biological testing are all ≥95% purity as determined by HPLC-MS and ^1^H NMR. ^1^H NMR experiments were recorded on Agilent DD2 400MHz spectrometers at ambient temperature. Samples were dissolved and prepared in deuterated solvents (CDCl_3_, CD_3_OD and DMSOd_6_) with residual solvents being used as the internal standard in all cases. All deuterated solvent peaks were corrected to the standard chemical shifts (CDCl_3_, dH = 7.26 ppm; CD_3_OD, dH = 3.31 ppm; DMSOd_6_, dH = 2.50 ppm). Spectra were all manually integrated after automatic baseline correction. Chemical shifts (d) are given in parts per million (ppm), and coupling constants (J) are given in Hertz (Hz). The proton spectra are reported as follows: d (multiplicity, coupling constant J, number of protons). The following abbreviations were used to explain the multiplicities: app = apparent, b = broad, d = doublet, dd = doublet of doublets, ddd = doublet of doublet of doublets, dddd = doublet of doublet of doublet of doublets, m = multiplet, s = singlet, t = triplet. All samples were analyzed on Agilent 1290 series HPLC system comprised of binary pumps, degasser and UV detector, equipped with an auto-sampler that is coupled with Agilent 6150 mass spectrometer. Purity was determined via UV detection with a bandwidth of 170nm in the range from 230-400nm. The general LC parameters were as follows: Column - Zorbax Eclipse Plus C18, size 2.1 X 50 mm; Solvent A: 0.10 % formic acid in water, Solvent B: 0.00 % formic acid in acetonitrile; Flow rate – 0.7 mL/min; Gradient: 5 % B to 95 % B in 5 min and hold at 95 % B for 2 min; UV detector – channel 1 = 254 nm, channel 2 = 254 nm. Mass detector Agilent Jet Stream – Electron Ionization (AJS-ES).

The following abbreviations are used:

THF: tetrahydrofuran

DCM or CH_2_Cl_2_: dichloromethane

DCE: dichloroethane

NaHCO_3_: sodium bicarbonate

HCl: hydrogen chloride

MgSO_4_: magnesium sulfate

Na_2_SO_4_ : sodium sulfate

DME: dimethoxyethane

n-BuLi: n-butyllithium

DMF: dimethylformamide

DMSO: dimethylsulfoxide

Et_2_O: diethyl ether

MeOH: methanol

EtOAc: ethyl acetate

*tert-butyl 3-cyano-3-(3-(trifluoromethyl)benzyl)pyrrolidine-1-carboxylate* (**2**): To a stirring, N_2_ purged, solution of diisopropylamine (1.60 mL, 11.42 mmol) in THF (30.0 mL) in a flame dried 100 mL RBF at -78 ^o^C, was added a 2.5 M solution of n-BuLi (4.60 mL, 11.5 mmol) in hexanes dropwise over 5 min. After stirring for 10 min, was added tert-butyl 3-cyanopyrrolidine-1-carboxylate **1** (2.04 g, 10.41 mmol) in 10 mL of THF over 5 min by addition funnel. After stirring 1 h, was added 3-trifluoromethylbenzyl bromide (1.90 mL, 12.4 in 10 mL of THF over 6 min by addition funnel. After 1.5 h, reaction quenched upon addition of 20 mL of a saturated solution of NH_4_OH, the layers were separated, and the aqueous portion was extracted with CH_2_Cl_2_ (3X 15 mL). The combined organic portions were dried over Na_2_SO_4_ and concentrated under reduced pressure. Purification was accomplished by flash chromatography, eluting with gradients of 10% and 30% of EtOAc/Hexanes, collecting in 120 mL fractions. The product containing fractions (4-6) were concentrated under reduced pressure to obtain 2.84 g (77.1 %) of nitrile **2** as a colorless oil. (mixture of rotamers) ^1^H NMR (400 MHz, CDCl_3_) ^1^H NMR (400 MHz, DMSO) δ 7.68 (s, 2H), 7.64 (s, 2H), 3.58 (d, *J* = 11.1 Hz, 1H), 3.55 – 3.45 (m, 1H), 3.40 (dd, *J* = 11.4, 6.4 Hz, 1H), 3.36 – 3.28 (m, 26H), 3.23 – 3.08 (m, 2H), 2.20 – 2.11 (m, 2H), 1.40 (s, 9H). ^19^F NMR (376 MHz, DMSO) δ -61.20 (d, *J* = 16.0 Hz). ESI-MS (m/z): 299.1 [M-C­_4_H_9_+1].

*tert-butyl 3-(aminomethyl)-3-(3-(trifluoromethyl)benzyl)pyrrolidine-1-carboxylate* (**3**): To a stirring solution of nitrile **2** (2.64 g, 7.44 mmol) in freshly distilled EtOH (13.0 mL) in a 50 mL RBF at room temperature, was added Raney-Nickel (41 mg). The flask was evacuated then back-filled with 1 atm of H_2_ gas. After 16 h, the slurry filtered over celite, washing with CH_2_Cl_2_. The supernatant was collected and concentrated under reduced pressure. Purification was accomplished by flash chromatography, eluting with solvent gradient of 5% and 10% MeOH/ CH_2_Cl_2_, collecting in 120 mL fractions. The product containing fractions (5-7) were concentrated under reduced pressure to obtain amine **3** (2.43 g, 95.4 %) as a colorless oil. (mixture of rotamers) ^1^H NMR (400 MHz, DMSO) δ 7.53 (s, 2H), 7.49 (s, 2H), 3.23 – 3.11 (m, 2H), 3.08 – 2.93 (m, 2H), 2.75 (s, 2H), 2.31 (s, 2H), 1.70 – 1.49 (m, 4H), 1.39 – 1.30 (m, 9H). ^19^F NMR (376 MHz, DMSO) δ -61.20 (d, *J* = 16.0 Hz). ESI-MS (m/z): 359.2 [M+1].

*tert-butyl 3-(((4-(((tert-butyldimethylsilyl)oxy)methyl)-3-nitrophenyl)amino)methyl)-3-(3-(trifluoromethyl)benzyl)pyrrolidine-1-carboxylate* (**4**): To a stirring solution of amine **3** (2.34 g, 6.54 mmol) in toluene (22.0 mL) at room temperature, added ((4-bromo-2-nitrobenzyl)oxy)(tert-butyl)dimethylsilane (2.43 g, 7.02 mmol), Cs_2_CO_3_ (6.54 g, 20.0 mmol) and (±)-BINAP (206.1 mg, 0.331 mmol). A stream of N_2_ gas was bubbled through the solution for 5 minutes, after which time Pd(OAc)_2_ (73.6 mg, 0.328 mmol). The reaction was heated to reflux for 18 hours under positive N_2_, cooled to room temperature, diluted with CH_2_Cl_2_, filtered over celite and the celite pad was washed with CH_2_Cl_2_. The resulting crude solution was concentrated under reduced pressure to yield a brown oil. Purification was accomplished by flash chromatography, eluting with gradients of 5% and 15% EtOAc/hexanes, collecting in 120 mL fractions. The product containing fractions (6-9) were concentrated under reduced pressure to obtain 2.23 g (54.7 %) of aniline **4** as a red oil. ^1^H NMR (400 MHz, CDCl_3_) δ 7.63 (d, *J* = 8.2 Hz, 1H), 7.51 (d, *J* = 7.2 Hz, 1H), 7.41 (t, *J* = 7.5 Hz, 1H), 7.37 (s, 1H), 7.31 (d, *J* = 7.4 Hz, 1H), 6.85 (dd, *J* = 8.4, 1.2 Hz, 1H), 4.98 (s, 2H), 3.45 (s, 2H), 3.36 (s, 2H), 3.02 (s, 2H), 2.88 (s, 2H), 1.89 (td, *J* = 13.4, 6.7 Hz, 1H), 1.82 (td, *J* = 13.3, 6.7 Hz, 1H), 1.47 (s, 8H), 0.95 (s, 8H), 0.12 (s, 5H). ^19^F NMR (376 MHz, CDCl_3_) δ -62.71. ESI-MS (m/z): 646.3 [M+23].

*tert-butyl 3-(((4-(hydroxymethyl)-3-nitrophenyl)amino)methyl)-3-(3-(trifluoromethyl)benzyl)pyrrolidine-1-carboxylate* (**5**): To a stirring solution of silyl ether **4** (2.19 g, 1.45 mmol) in THF (10.0 mL) in a flame dried 100 mL RBF at 0 ^o^C, was added a 1.0 M Tetrabutylammonium Fluoride (4.2 mL) solution in THF dropwise over 5 min and the resulting reaction was allowed to warm to room temperature. After 2 h, 20 mL of brine and 15 mL of EtOAc were added to quench the reaction. Layers were separated, and the aqueous portion was extracted with EtOAc (2X 20 mL). The combined organic layers were dried over Na_2_SO_4_ and concentrated under reduced pressure. Purification was accomplished by flash chromatography, eluting with solvent gradients of 20% and 40% of EtOAc/Hexanes, collecting in 120 mL fractions. The product containing fractions (3-6) were concentrated under reduced pressure to obtain 1.45 g (81.1 %) of alcohol **5** as an orange oil. ^1^H NMR (400 MHz, CDCl_3_) δ 7.51 (d, *J* = 7.1 Hz, 1H), 7.43 – 7.37 (m, 2H), 7.34 (s, 1H), 7.28 (d, *J* = 7.9 Hz, 1H), 7.21 (d, *J* = 2.4 Hz, 1H), 6.80 (dd, *J* = 8.3, 2.4 Hz, 1H), 4.76 (s, 2H), 3.58 – 3.40 (m, 2H), 3.36 (d, *J* = 11.2 Hz, 1H), 3.32 – 3.15 (m, 1H), 3.01 (s, 2H), 2.93 – 2.80 (m, 2H), 1.93 – 1.75 (m, *J* = 19.6, 12.8, 6.5 Hz, 2H), 1.44 (d, *J* = 8.8 Hz, 9H). ESI-MS (m/z): 454.2 [M-C­_4_H_9_+1].

*(2-nitro-4-(((3-(3-(trifluoromethyl)benzyl)pyrrolidin-3-yl)methyl)amino)phenyl)methanol* (**6**): To a stirring solution of tertbutyl carbamate **5** (1.04 g, 2.04 mmol) in CH_2_Cl_2_ (9.0 mL) in a flame dried 50 mL RBF at -10 ^o^C, apparatus was flushed with N_2_ before boron trifluoride etherate (1.00 mL, 8.10 mmol) was added one portion and the resulting reaction was allowed to slowly warm to room temperature. After 4 h, 15 mL of a saturated NaHCO_3_ solution was added quench the reaction. The layers were separated, and the aqueous portion was extracted with a 3:1 mixture of isopropanol and CHCl_3_ (6X 15 mL). The combined organic layers were dried over Na_2_SO_4_ and concentrated under reduced pressure. Purification was accomplished by flash chromatography, eluting with solvent gradients of 5% and 20% 1 M NH_4_OH in MeOH/CH_2_Cl_2_, collecting in 22 mL fractions. The product containing fractions (2-4) were concentrated under reduced pressure to obtain 246.5 mg (29.5 %) of amine **6** as an orange oil. ^1^H NMR (400 MHz, cd_3_od) δ 7.42 (d, *J* = 8.6 Hz, 1H), 7.25 – 7.12 (m, 6H), 6.92 (dd, *J* = 8.5, 2.4 Hz, 1H), 4.74 (s, 2H), 2.99 – 2.88 (m, *J* = 22.9 Hz, 3H), 2.87 – 2.78 (m, *J* = 17.6, 4.1 Hz, 2H), 2.74 (d, *J* = 11.5 Hz, 1H), 1.90 – 1.77 (m, 1H), 1.76 – 1.60 (m, 1H). ^19^F NMR (376 MHz, DMSO) δ -63.45. ESI-MS (m/z): 410.2 [M+1].

 (4-(((1-((1H-indol-3-yl)methyl)-3-(3-(trifluoromethyl)benzyl)pyrrolidin-3-yl)methyl)amino)-2-nitrophenyl)methanol (**CIDD-0149830**): To a stirring solution of amine **6** (1 equiv.) in 1,2-DCE (0.1 M) was added 1H-indole-3-carbaldehyde (1.5 equiv.). After stirring for 1 hour at room temperature, NaBH(OAc)_3_ (2.5 equiv) was added. The reaction was allowed to stir for 24 hours, at which point, reaction was quenched upon addition of 1.0 ml of saturated NaHCO_3_ and 1.0 mL of EtOAc. Layers were separated then the aqueous portion was extracted EtOAc (3 x 10 mL). Combined organic fractions, dried over Na_2_SO_4_, and concentrated under reduced pressure. Purification of the crude product was carried out via by flash chromatography to afford the desired compound **CIDD-0149830** as an Orange oil: 7.4 mg, 39.0%. ^1^H NMR (400 MHz, cd_3_od) δ 7.64 (d, *J* = 7.8 Hz, 1H), 7.48 (d, *J* = 11.6 Hz, 2H), 7.42 (d, *J* = 8.5 Hz, 1H), 7.40 – 7.31 (m, 3H), 7.17 (d, *J* = 2.4 Hz, 1H), 7.13 (d, *J* = 8.1 Hz, 1H), 7.08 (t, *J* = 7.0 Hz, 1H), 6.87 (dd, *J* = 8.5, 2.5 Hz, 1H), 4.75 (m, 2H), 3.17 (ABq, *ΔV*_AB_ = 12.4 Hz, *J_AB_* = 13.6 Hz, 2H), 3.15 (s, 1H), 2.96 (s, 13H), 2.11 – 2.01 (m, 5H), 1.81 – 1.68 (m, 1H). ^19^F NMR (376 MHz, DMSO) δ -64.15. ESI-MS (m/z): 539.2 [M+1].
